# Autophagy modulation effect on homotypic transfer of intracellular components via tunneling nanotubes in mesenchymal stem cells

**DOI:** 10.1186/s13287-024-03813-1

**Published:** 2024-07-02

**Authors:** Fatemeh Sadeghsoltani, Çığır Biray Avci, Parisa Hassanpour, Sanya Haiaty, Mohamad Rahmati, Ali Mota, Reza Rahbarghazi, Maryam Nemati, Mahdi Mahdipour, Mehdi Talebi, Leila Sabour Takanlou, Maryam Sabour Takanlou, Amir Mehdizadeh

**Affiliations:** 1 Stem Cell Research Center, Tabriz University of Medical Sciences, Tabriz, Iran; 2 Department of Clinical Biochemistry and Laboratory Medicine, School of Medicine, Tabriz University of Medical Sciences, Tabriz, 5166614766 Iran; 3 Department of Medical Biology, Faculty of Medicine, Ege University, Izmir, Turkey; 4 Infectious and Tropical Diseases Research Center, Tabriz University of Medical Sciences, Tabriz, Iran; 5 Department of Applied Cell Sciences, Faculty of Advanced Medical Sciences, Tabriz University of Medical Sciences, Tabriz, 5166653431 Iran; 6 Department of Genetic, Tabriz Branch, Islamic Azad University, Tabriz, Iran; 7 Hematology and Oncology Research Center, Tabriz University of Medical Sciences, Tabriz, Iran

**Keywords:** Mesenchymal stem cells, Autophagy, Tunneling nanotubes, Mitochondrial donation, Apoptosis, Wnt signaling pathway

## Abstract

**Background:**

Recent studies have proved the role of autophagy in mesenchymal stem cell (MSCs) function and regenerative properties. How and by which mechanism autophagy modulation can affect the juxtacrine interaction of MSCs should be addressed. Here, the role of autophagy was investigated in the formation of tunneling nanotubes (TNTs) and homotypic mitochondrial donation.

**Methods:**

MSCs were incubated with 15 µM Metformin (Met) and/or 3 µM 3-methyladenine (3-MA) for 48 h. The formation of TNTs was assessed using bright-field and SEM images. The mitochondria density and ΔΨ values were monitored using flow cytometry analysis. Using RT-PCR and protein array, the close interaction and shared mediators between autophagy, apoptosis, and Wnt signaling pathways were also monitored. The total fatty acid profile was assessed using gas chromatography.

**Result:**

Data indicated the increase of TNT length and number, along with other cell projections after the induction of autophagy while these features were blunted in 3-MA-treated MSCs (*p* < 0.05). Western blotting revealed the significant reduction of Rab8 and p-FAK in 3-MA-treated MSCs (*p* < 0.05), indicating the inhibition of TNT assembly and vesicle transport. Likewise, the stimulation of autophagy increased autophagic flux and mitochondrial membrane integrity compared to 3-MA-treated MSCs. Despite these findings, protein levels of mitochondrial membrane Miro1 and 2 were unchanged after autophagy inhibition/stimulation (*p* > 0.05). We found that the inhibition/stimulation of autophagy can affect the protein, and transcription levels of several mediators related to Wnt and apoptosis signaling pathways involved in different cell bioactivities. Data confirmed the profound increase of mono and polyunsaturated/saturated fatty acid ratio in MSCs exposed to autophagy stimulator.

**Conclusions:**

In summary, autophagy modulation could affect TNT formation which is required for homotypic mitochondrial donation. Thus, the modulation of autophagy creates a promising perspective to increase the efficiency of cell-based therapies.

**Supplementary Information:**

The online version contains supplementary material available at 10.1186/s13287-024-03813-1.

## Introduction

During the last decades, the discovery and application of various progenitors and stem cell types have revolutionized human medicine [1, 2]. Mesenchymal stem cells (MSCs) are the most commonly used stem cells in several experiments and human clinical trials [3–7]. These cells can use a multitude of mechanisms to exert their regenerative properties in the injured sites. Secretion of chemokines, cytokines, growth factors, metabolic products, and extracellular matrix, commonly referred to as the paracrine effect, is the main underlying mechanism for reparative outcomes [8]. In stem cell secretome, various extracellular vesicle (EVs) types, including exosomes and microvesicles, can transfer signaling molecules and maintain reciprocal communication between cells [9, 10].

Tunneling nanotubes (TNTs) are cytoskeletal protrusions and maintain juxtacrine cell-to-cell communication [11]. TNTs enable the transmission of intracellular organelles in bi- and uni-directional manners, exhibiting distinct structural and functional characteristics in various cell types [12]. These nano-sized structures are supported by the actin polymer backbone and can generated via the elongation of cellular protrusion and dislodgement [13]. Of note, TNTs can facilitate the transfer of diverse cellular cargo such as mitochondria and Golgi vesicles, as well as vesicles, genetic molecules like microRNA and siRNA, proteins, and ions between the cells [14]. It has been found that TNTs can also distribute various microbial pathogens and factors associated with several pathological conditions such as neurological disorders and cancers [15]. MSCs can generate TNTs as intercellular bridges to transport cargo, i.e. mitochondria, for regenerative purposes [16, 17]. Along with cytoskeletal remodeling, several enzymes and mediators such as Miro1 and 2 (mitochondrial Rho-GTPases), Rab8, TRAK1, TRAK2, and Myo19 are required for organelle donation. It has been found that knockdown of Miro1 inhibits TNT production and blunt regenerative potential of MSCs [17, 18].

Autophagy serves as a crucial intracellular degradation system and is regulated by various autophagy-related (ATG) proteins. By the activation of autophagy, the host cells can recycle abnormal cytoplasmic components, damaged and senescent organelles, and misfolded protein aggregates [19]. Emerging data have proved that different cells bioactivate after the activation of autophagy. For instance, paracrine activity, cytoskeletal remodeling, migration capacity, etc. are modulated by autophagy response [20–24].

Here, we aimed to assess the possible role of autophagy response on TNT formation in MSCs and organelle donation. It is thought that the data from the present study can help us in the elucidation of the relationship between autophagy status, TNT formation, and organelle donation in MSCs.

## Materials and methods

### MSC culture and expansion protocol

MSC line (Cat no: IBRC-C10680) was obtained from the Iranian Biological Resource Center (Iran) and expanded in culture plates with low-glucose content Dulbecco’s modified Eagle’s Medium (DMEM/LG; Gibco) at standard conditions (37 °C, 95% relative humidity, and 5% CO_2_). The culture medium was supplemented with 10% fetal bovine serum (FBS, Gibco) and 1% Penicillin/Streptomycin (Biosera) solutions, and renewed at regular intervals of 3–4 days. Cells with a confluence level of 70–80% were subcultured using 0.25% Trypsin-EDTA solution. Cells of passages 3–6 were utilized for different analyses.

### MTT assay

To evaluate the effect of autophagy modulation on survival rate, MSCs were exposed to Metformin (Met; Cat no:1115-70-4) and 3-Methyl Adenine (3-MA; Cat no: CS-5207) respectively for 48 h. Thereafter, the conventional MTT colorimetric method was performed to find the maximum chemical content with the least toxic effect. In this regard, MSCs (~ 1 × 10^4^) were placed in each well of 96-well plates (SPL, Korea), cultured at standard conditions, and allowed to reach 70–80% confluence. Then, cells were exposed to different concentrations of Met (1, 5, 15, 20, 50, 75 µM) for 48 h. After incubation time, supernatants were removed 200 µl of 5 mg/ml MTT solution (Sigma-Aldrich; Cat no: M5655) was overlaid on each well, and plates were maintained at 37 °C for the next 4 hours. Then, supernatants were removed and replaced with 100 µl dimethyl sulfoxide (DMSO; Merck). The OD of different groups was read using a microplate reader (BioTek, USA) and the viability of cells was calculated related to the non-treated control group. The inhibition of autophagy was performed using 3 µM 3-MA according to previously published data.

### Monitoring of TNT compartments

The formation of TNTs was evaluated using an inverted microscope and scanning electron microscopy (SEM). To this end, MSCs treated with 15 µM Met and 3 µM 3-MA for 48 h, and were fixed using a 1% paraformaldehyde (PFA) solution. Serial high-power fields were monitored using an inverted microscope, and the number and length of TNTs were measured using AxioVision 4.8.2 software. For SEM analysis, MSCs from different groups were dehydrated using an ascending EtOH series, gold-sputtered, and imaged using an SEM instrument (Model: MIRA3 TESCAN; Czech Republic).

### Immunofluorescence imaging

LysoTracker and MitoTracker staining were performed to assess the stimulatory and inhibitory effects of Met and 3-MA on later stages of autophagy and mitochondrial membrane integrity, respectively. To this end, MSCs were seeded in 4-well Chambered Cell Culture Slides (SPL) at a density of 10^4^ cells per well and kept under standard conditions. After 24 h, MSCs were treated with 15 µM Met and 3 µM 3-MA for 48 h, washed with pre-cold PBS, and stained with 200 µM LysoTracker Green (cat no: L7526, Sigma-Aldrich) and/or 20 nM Green Mito Tracker solution (Cat no: M7514) for 1 h. After three times washing with PBS, cell nuclei were stained with a 1 µg/ml DAPI (Sigma-Aldrich) solution for 30 s. The cells were imaged using the BX41 Olympus immunofluorescence microscope.

### Monitoring mitochondrial uptake

Non-treated MSC mitochondria were extracted upon reaching the 70–80% confluence. Cells were detached using Trypsin-EDTA solution and were centrifuged at 400 g for 15 min. Cell pellets were incubated with 1X, 2.5X, and hypo-osmotic lysis buffers. The solutions were centrifuged at 17,000 g for 20 min at 4 °C to collect the mitochondrial pellets and stained with 20 nM Green Mito Tracker solution for 1 h. MSCs pre-treated with 15 µM Met and 3 µM 3-MA were incubated with labeled mitochondria for 48 h. After three PBS washes, the cells were detached and re-suspended in 500 µl PBS solution and analyzed using the BD^®^ FACSCalibur system and FlowJo software (Ver.7.6.1).

### Western blotting analysis

Western blotting was used to measure protein associated with autophagy (BCLN-1, LC3, and P62), mitochondrial donation (Miro-1, Miro-2, and Rab8), and cytoskeletal remodeling [focal adhesion kinase (FAK)]. MSCs were lysed using protein lysis buffer (NP40, Tris-HCl, EDTA, NaCl, Sodium Deoxycholate, and sodium dodecyl sulfate), and centrifuged at 12,000 g for 30 min at 4 °C. Using 10% SDS-PAGE gel electrophoresis, proteins were separated and then transferred onto PVDF membranes. Membranes were blocked using 2% skim milk solution at RT for 1 h, and incubated with primary antibodies overnight at 4 °C as follows; BECLN-1 (Cat no: sc-48,341; Santa Cruz Biotechnology, Inc), LC3 (Cat no: 2775; Cell Signaling Technology, Inc), and P62 (Cat no: sc-10,117; Santa Cruz Biotechnology, Inc), Miro1 (Cat no: sc-398,520; Santa Cruz Biotechnology, Inc), Miro 2 (Cat no: sc-135,387; Santa Cruz Biotechnology, Inc), Rab8 (Cat no: sc-81,909; Santa Cruz Biotechnology, Inc), (Cat no: sc-81,493; Santa Cruz Biotechnology, Inc). After that, membranes were washed with PBTS and incubated with the secondary HRP-conjugated secondary antibodies (Cat no: sc-2357; Santa Cruz Biotechnology, Inc). Using an ECL solution, immunoreactive bands were detected on the X-ray films. The band density of each immunoblot was measured using ImageJ software (NIH; Ver 1.4.) related to an internal housekeeping β-actin (Cat no: sc-47,778 Santa Cruz Biotechnology, Inc). This experiment was performed in triplicate.

### PCR array

The expression of different mediators related to autophagy and Wnt signaling pathways was monitored using PCR array analysis. To this end, treated MSCs were lysed using the RNA extraction kit Trizol Reagent (Cat no: 302-001; RiboExLs), and sample purity was measured by the Picodrop system. The isolated RNAs were reverse-transcribed into cDNA using a SMOBIO cDNA Synthesis Kit [RP1400] ExcelRT™ Reverse Transcription Kit II, 100 Rxn]. After cDNA synthesis, the expression of all genes was determined by using Human Autophagy RT^2^ Profiler PCR Array (Cat no: PAHS-084Z; Qiagen GeneGlobe), Human Wnt RT^2^ Profiler PCR Array (Cat no: PAHS-043ZF; Qiagen GeneGlobe), and Light Cycler 480 Instrument II (Roche). Raw data were processed using the 2^−ΔΔCT^ formula to calculate fold changes of each gene. Transcription rates more than 2-fold were considered as the cut-off value with three sets of experiments.

### Protein array

To assess whether autophagy modulation can alter protein levels of the apoptosis signaling pathway, 43 pro- and anti-apoptosis proteins were monitored using a human apoptosis antibody array (Cat no: ab134001; Abcam) according to the manufacturer’s instructions. In brief, protein levels were measured using a BCA Protein Quantification Kit (Cat no: A101251; Parstous). Then, recommended protein concentrations were added to membranes with capturing antibodies spotted in arrays and kept at 4 °C overnight. After that, samples were washed several times with a washing buffer and blocked using a blocking buffer. The procedure was continued with the addition of biotin-conjugated anti-cytokines and HRP-streptavidin. The membranes were carefully washed and immunoreactive spots were detected using the ECL solution Western Blot Imaging System (Model: SB-14,007; Sabz Biomedicals). The density of each spot was measured using ImageJ software (NIH; ver. 1.4). In this study, protein levels more than 2-fold were considered as the cut-off values.

### Total fatty acid profile assays

The fatty acid profile was monitored using gas chromatography via direct trans-esterification method as previously elucidated [25]. The pooled cells (~ 1 × 10^6^ cells) were subjected to transesterification to convert their methyl esters by introducing 200 µl of acetyl chloride reagent in 2 mL methanol-hexane (4:1, v/v), and methanolysed at 100 °C for 1 h. After the addition of a 6% solution of K_2_CO_3_, the upper phase of hexane was collected and used for analysis. The methyl ester pattern was identified through Teknokroma TR CN100 column (60 × 0.25 mm) using a Buck Scientific gas chromatograph (Model 610, SRI Instruments, Torrance, USA), and PeakSimple, version 3.59 (SRI Inc) relative to Tridecanoic acid (13:0) as an internal standard. The percentage of specific fatty acids, such as saturated fatty acids (SFAs; Myristate, Palmitate, Stearate, and Pentadecanoate), monounsaturated fatty acids (MUFAs; Oleate), and polyunsaturated fatty acids (PUFAs; Linoleate) were analyzed and expressed as a percentage of the total extracted fatty acids.

### Statistical analysis

Analysis of data was performed using the GraphPad PRISM (Ver. 8.4.3) and data (mean ± SD) were monitored using One-Way ANOVA with Tukey post hoc analysis. *p* < 0.05 was considered statistically significant.

## Results

### Met dose selection

**Fig. 1:**
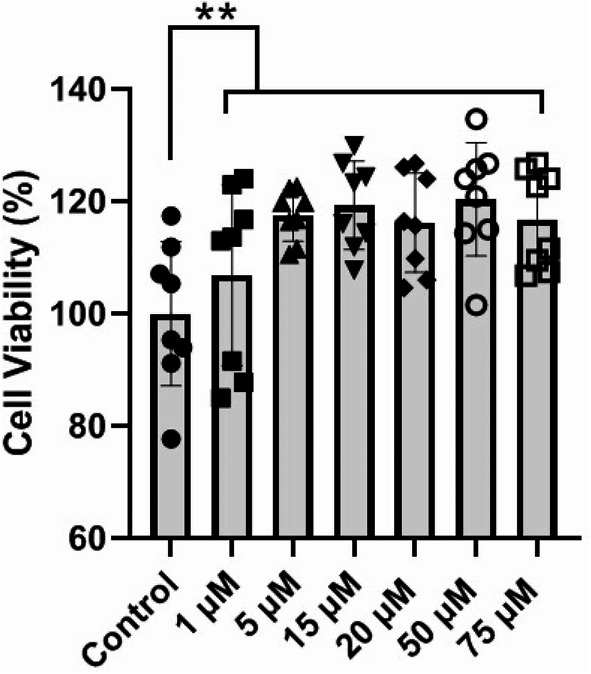
Monitoring MSC survival rate incubated with different doses of Met, 1, 5, 15, 20, 50, and 75 µM, after 48 h. Data indicated a significant increase in MSC viability in Met-treated groups compared to the control cells (*n* = 8). In this study, 15 µM Met was selected for other analyses. Data are expressed as mean ± SD. One-way ANOVA with Tukey post hoc. **p < 0.01

In this study, an MTT assay was used to select the maximum Met concentration with the minimum cytotoxicity (Fig. 1). Data indicated that the incubation of MSCs with different concentrations of Met, 1, 5, 15, 25, 50, and 75 µM for 48 h led to enhanced viability compared to the non-treated control (*p* < 0.01; Fig. 1). No statistically significant differences were obtained in terms of MSC survival between the Met-treated groups (*p* > 0.05). According to data from current experiments and previous studies, 15 µM Met was used for subsequent analyses. To inhibit autophagy, 3 µM 3-MA was used in different assays according to previous data.

### Autophagy stimulation promoted TNT formation in MSCs

**Fig. 2:**
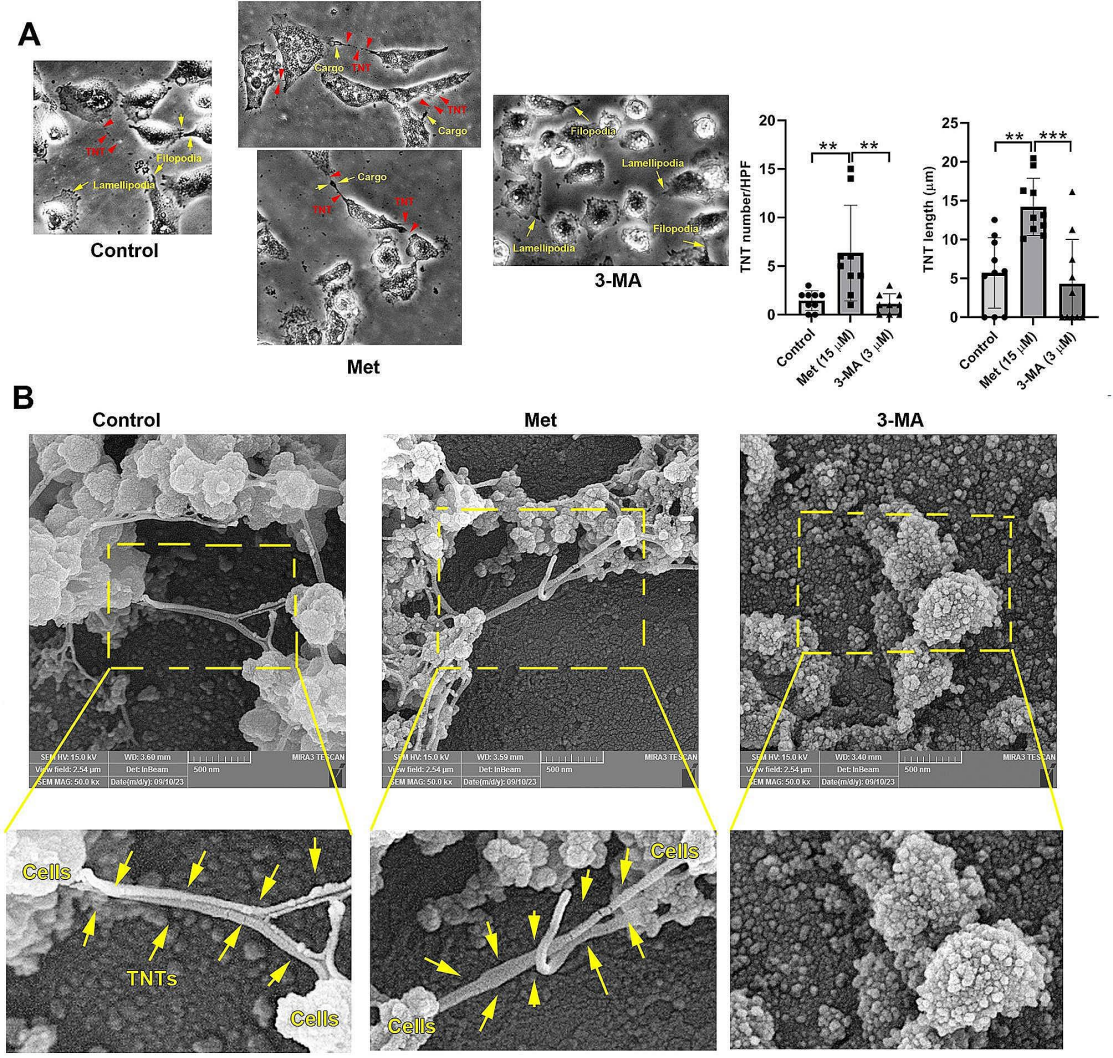
TNT formation and projections in MSCs exposed to 15 µM Met, and 3 µM 3-MA after 48 h. Bright-field images (**A**), and SEM images (**B**). Data indicated the formation of different cell projection types including TNTs (red arrows) filopodia, and lamellipodia (yellow arrows) in control MSCs (**A**). Data indicated that the number and length of TNTs (red arrowheads) were increased in Met-treated MSCs compared to the control and 3-MA groups. In Met-treated MSCs, intercellular transfer of cargo can be detected inside the TNTs (yellow arrows). Data showed the lack of significant differences in terms of TNT length and number in 3-MA treated MSCs compared to the control group. SEM images indicated the existence of TNT links between the control and Met-treated MSCs (**B**; yellow arrows). In the presence of 3-MA, MSCs lose the ability to produce TNT bridges. Data are expressed as mean ± SD. One-way ANOVA with Tukey post hoc. **p < 0.01; ***p < 0.001

To investigate the relationship between autophagy response, cell morphology and TNT formation, MSCs were treated with 15 µM Met and 3 µM 3-MA for 48 h and examined using bright-field and SEM images (Fig. 2A). Bright-field images indicated the formation of intercellular TNT connections in MSCs after being exposed to autophagy modulators (red arrowheads; Fig. 2A). Based on the results, Met statistically increased the number and length of TNTs compared to the control and 3-MA treated MSCs (*p* < 0.01; Fig. 2A). Data confirmed several dense granules, or nodules inside the TNTs (yellow arrows), indicating active transportation between the MSCs. In contrast to the Met-treated cells, the number and length of TNTs were statistically reduced in the 3-MA group with faint cytoskeletal remodeling, leading to almost rounded margins and a reduced number of cellular projections (TNTs, filopodia, and lamellipodia). These findings were also confirmed by SEM ultrastructural images (Fig. 2B). SEM images showed the TNT compartments between 2D cultured MSCs in control and Met-treated groups (yellow arrows). TNT structures were like bridges and strands which signified the active inter-MSC connections after autophagy stimulation (Fig. 2B). In the 3-MA treated MSCs, cells exhibited round-shape morphologies with reduced TNT units. These data indicate that the stimulation of autophagy can promote the MSC-to-MSC juxtacrine interaction via the activation of cytoskeletal remodeling and the formation of TNTs. In contrast, autophagy inhibition reduced TNT structures and cellular projections, leading to the reduction of physical contact between the cells.

### Met can induce autophagy flux inside the MSCs

**Fig. 3:**
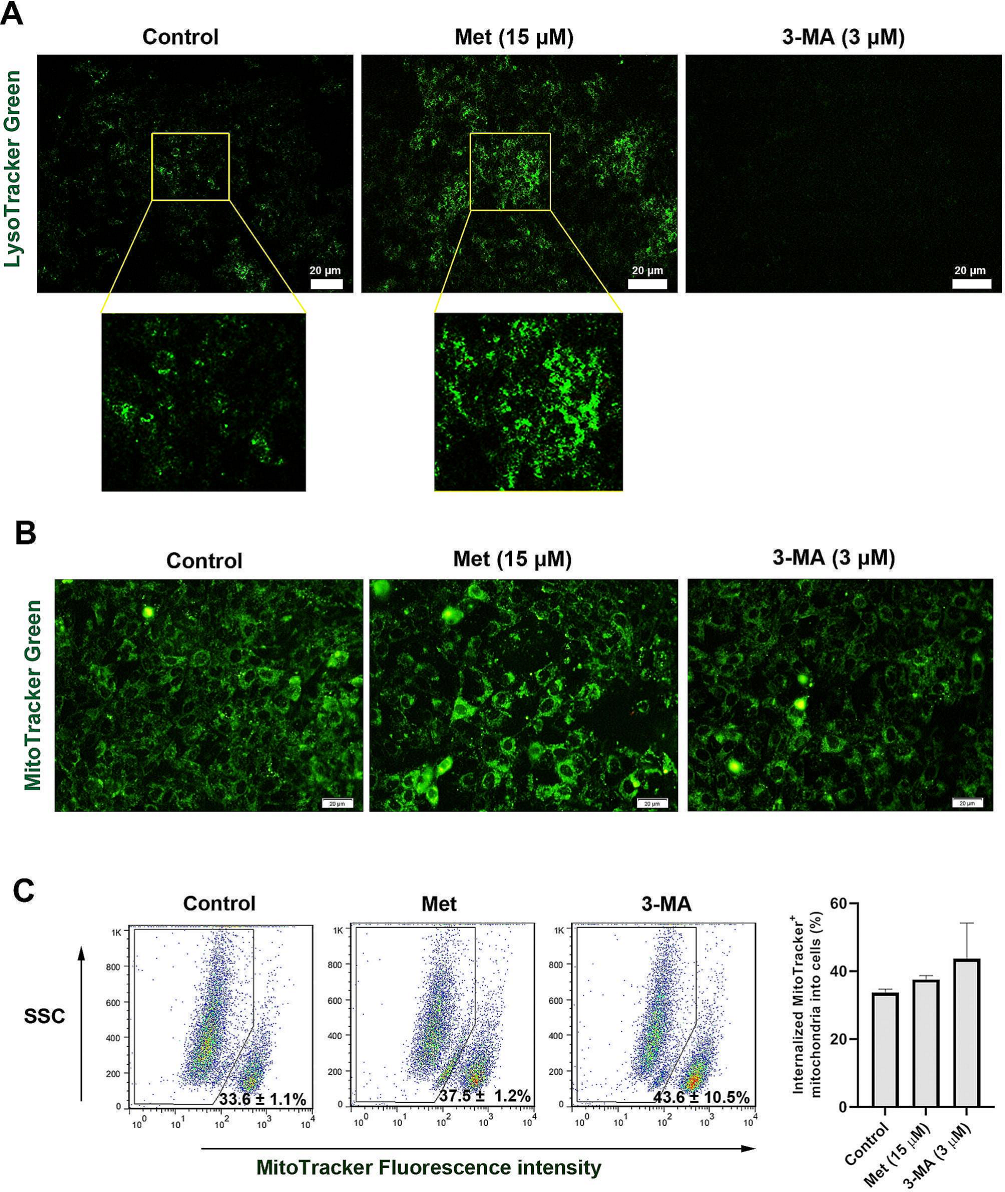
Monitoring autophagic process in 15 µM Met, and 3 µM 3-MA treated MSCs using LysoTracker staining after 48 h (**A**). Immunofluorescence (IF) images indicated the increase of acidic compartments and autophagy flux in the presence of Met compared to the control group. The number and intensity of green LysoTracker particles were at the minimum levels in 3-MA treated MSCs related to control cells. Mitochondrial membrane integrity was studied using Green Mito Tracker staining (**B**). IF showed higher fluorescence intensity and an increase of fluorescent particles in Met-treated MSCs compared to control and 3-MA groups. These features indicated an appropriate ΔΨ feature and mitochondrial number inside the MSCs after being exposed to Met. Flow cytometry analysis of mitochondria internalization in MSCs after treatment with Met and 3-MA. MSCs from different groups were incubated with Green Mito Tracker-stained mitochondria. Despite the increase of mitochondria uptake in 3-MA treated cells, no statistically significant differences were achieved in terms of fluorescence intensity. Data are expressed as mean ± SD. One-way ANOVA with Tukey post hoc

Autophagic flux was monitored in different groups using LysoTracker, an acidotropic dye (Fig. 3A). This compound is pH-dependent fluorescence and indicates the fusion of autophagosomes with lysosomes. According to our data, Met increased the intensity and number of intracellular LysoTracker green particles compared to the non-treated control MSCs. In the 3-MA treated group, intracellular LysoTracker green particles are barely detectable, indicating the reduction or inhibition of autophagy flux. Even, these features were reduced prominently in 3-MA groups less than that of control MSCs. These data indicate that Met can stimulate the autophagy flux and autophagolysosome formation while 3-MA decreases the fusion of lysosomes with autophagosome.

### Met increased mitochondrial functional activity in MSCs

To investigate the relationship between stimulation/inhibition of autophagy and mitochondrial membrane integrity, MitoTracker Green staining was used (Fig. 3B). Data indicated that the intensity of MitoTracker Green^+^ particles was increased in Met-treated MSCs compared to the control and 3-MA groups. While a relatively similar fluorescence intensity pattern was achieved for the control and 3-MA groups (Fig. 3B). These data demonstrate that higher intensity staining of MSCs with MitoTracker Green is associated with increased mitochondrial content and respiration rate. Based on our data, the inhibition of autophagy did not alter the functional activity of mitochondria compared to the non-treated MSCs.

### Autophagy inhibition can alter mitochondrial internalization in MSCs

To investigate whether autophagy inhibition/stimulation is involved in mitochondrial reception and internalization, the isolated mitochondria were pre-labeled with MitoTracker Green, added to the supernatant, and internalization rate was analyzed using flow cytometry after 24 h. Data indicated that the number of internalized mitochondrial particles was relatively similar in the control and Met-treated MSCs in which 33.6 ± 1.1, and 37.5 ± 1.2% of MSCs were MitoTracker Green positive, respectively (Fig. 3C). These features were increased in 3-MA-treated MSCs and reached 43.6 ± 10.5%. Despite the lack of significant changes between the 3-MA-treated cells with control and Met groups, it can be said that probably the number and rate of mitochondrial internalization increased after autophagy inhibition.

### Met and 3-MA changed the protein levels related to TNT assembly

**Fig. 4:**
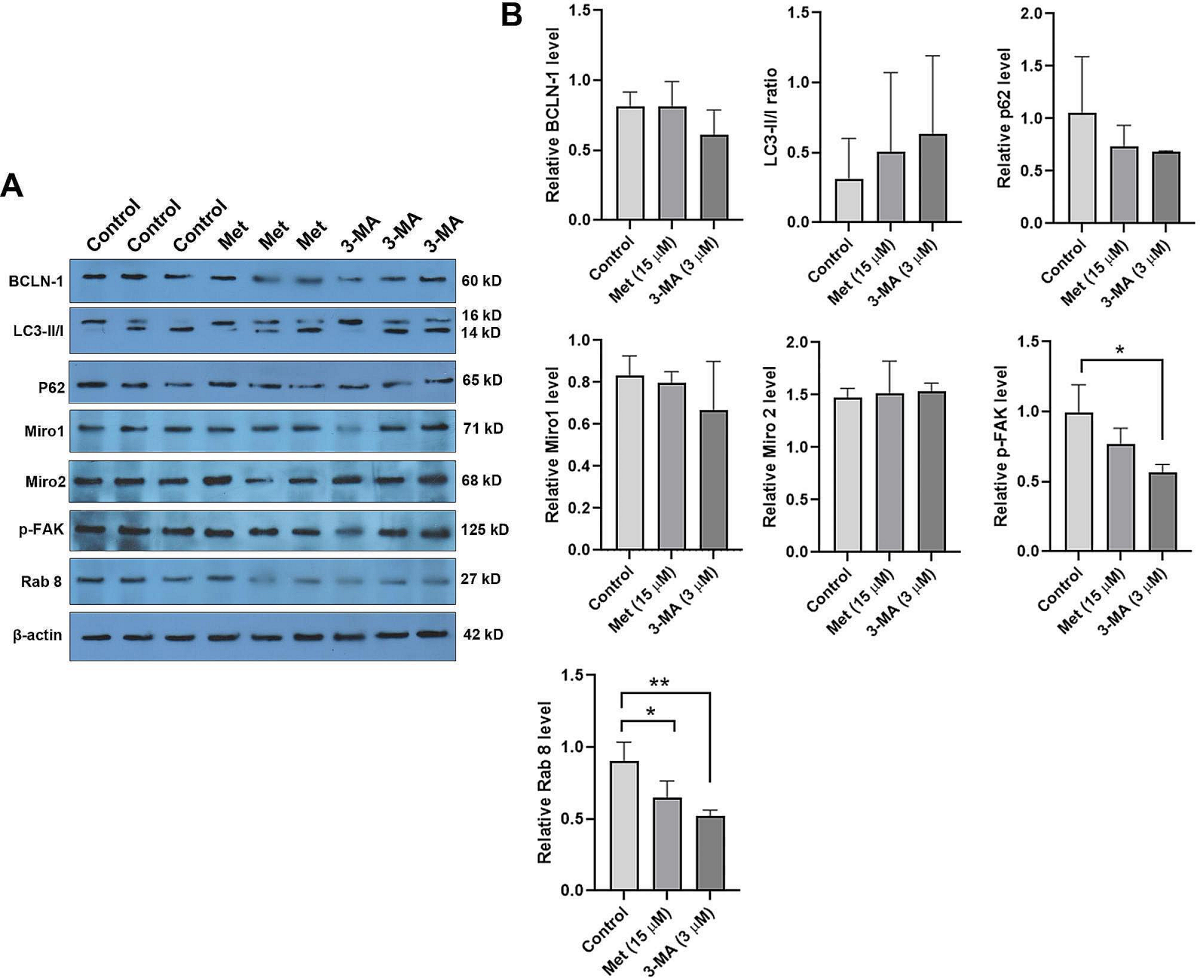
Western blotting (A and B). Data revealed the lack of significant differences in protein levels of autophagy machinery (BCLN1, LC3-II/LC3-I ratio, and p62) in the presence of 15 µM Met, and 3 µM 3-MA after 48 h. Protein levels of Miro1 and 2 remained unchanged in the presence of Met, and 3-MA. The levels of Rab8 and p-FAK related to TNT assembly and vesicle transport were reduced significantly in 3-MA-treated MSCs. Data are expressed as mean ± SD. One-way ANOVA and Tukey post hoc test (*n* = 3). *p < 0.05; **p < 0.01

In this study, western blotting was used to monitor protein levels of BCLN1, LC3-II/LC3-I ratio, and p62 in MSCs exposed to 3-MA and Met (Fig. 4A and **B**). According to the data, despite the reduction of BCLN1 in 3-MA treated MSCs, no statistically significant differences were obtained compared to the control and Met groups (*p* > 0.05). Protein levels of p62 and LC3-II/LC3-I ratio were not statistically significant in Met and 3-MA groups compared to the non-treated MSCs (*p* > 0.05). Data confirmed the lack of significant changes in protein levels of Miro1 and 2 in treated MSCs related to the control group (*p* > 0.05; Fig. 4A and **B**). Of note, protein levels of factors involved in TNT assembly were significantly changed in the presence of 3-MA as compared to the control MSCs (*p* < 0.05; Fig. 4A and **B**). Data indicated a significant reduction of GTPase Rab8 and p-FAK in MSCs exposed to 3-MA after 48 h. These data indicated that autophagy inhibitor can reduce protein levels of factors involved in TNT assembly and vesicle transportation.

### Met and 3-MA altered the expression of genes related to the autophagy signaling pathway

To monitor the expression of ATGs, the transcription of different genes was monitored using PCR array analysis (Table 1). Data showed that the expression of specific genes was changed in the presence of 15 µM Met and 3 µM 3-MA after 48 h. The expression of genes related to vacuole formation, ATG12 (3.31-fold), ATG16L1 (2.29-fold), ATG4C (2.73-fold), ATG5 (2.86-fold), ATG9B (2.69-fold) were up-regulated in Met-treated MSCs. We noted that the expression of other genes from the same signaling transduction such as ATG4A (2.89-fold), ATG4B (4.11-fold), ATG9A (2.89-fold), GABARAP (2.33-fold), GABARAPL2 (2.87-fold), MAP1LC3B (4.11-fold) was also induced MSCs after being exposed to the 3-MA. Based on the data, ATG4C, ATG4A, and ATG4B with proteolytic activity can foster the autophagy flux via interaction with GABARAP to fuse autophagosomes with lysosomes (Supplementary Table 1). According to the obtained data, 3-MA and Met can differently stimulate the expression of genes related to protein transport signaling transduction pathway in which the expression of ATG4A, ATG4B, ATG9A, GABARAP, GABARAPL2, and RAB24 (5.03-fold), ATG10 (1468.37-fold) was induced after 3-MA treatment whereas Met up-regulated the expression of ATG16L1, ATG4C, and ATG10 (467.23-fold). Data confirmed that the expression of shared genes between the autophagy and apoptosis pathway such as ATG12, ATG5, BAD (11.22-fold), EIF2AK3 (8.16-fold), HDAC1 (10.76-fold), HTT (107.49-fold), PTEN (9.37-fold) and TP53 (16.66-fold) was up-regulated in Met-treated MSCs compared to the non-treated control MSCs. Of note, CXCR4 (6.02-fold), TNF (4.06-fold), MAPK8 (2.64-fold), and ACTB (2.28) genes were up-regulated by 3-MA in MSCs from the same signaling pathway. We noted that both Met and 3-MA triggered the CASP3 (4.68-, and 3.97-fold), INS (14.01, and 9.06-fold), SNCA (7.77, and 6.19-fold), SQSTM1 (18.10, and 9-fold), associated with co-regulators of apoptosis and autophagy signaling transduction pathway. The transcription of genes related to intracellular signals like CTSD (4.16-fold), PIK3R4 (19.13-fold), RPS6KB1 (4.05-fold), and TMEM74 (4.85-fold) was also stimulated by Met. A similar trend was achieved in terms of CTSS (4.56-fold) and PIK3C3 (3.61-fold) expression in the 3-MA group. However, Met and 3-MA can activate common genes the ULK2 (11.54-, and 33.13-fold), and GAA (19.81-, and 3.25-fold), respectively from the same signaling axis. We found that the activation of genes associated with cell cycle such as PTEN, TP53, and RB1 (3.13-fold) after the modulation of autophagy by Met. Interestingly, the activity of genes related to chaperone-mediated autophagy (CMA) namely HSPA8 (26579.01-, and 7.89-fold) was induced in both Met and 3-MA groups. However, these values were more prominent in the Met-treated MSCs compared to the 3-MA group. The expression of EIF2AK3 (8.16-fold) belonging to pathogen-mediated autophagy response signaling was changed in the presence of Met while 3-MA was neutral to change the expression of this gene. These data demonstrate that Met and 3-MA can modulate the expression of various genes related to different signaling transduction pathways for autophagic activity.

**Table 1:** PCR array analysis of human autophagy signaling pathway in MSCs exposed to Met and 3-MA

Gene	Met	3-MA	Gene	Met	3-MA
*AKT1*	1.09	0.87	*GABARAPL2*	1.46	**2.87**
*AMBRA1*	1.69	0.45	*HDAC1*	**10.76**	1.69
*APP*	0.86	0.85	*HDAC6*	0.36	1.67
*ATG10*	**467.23**	**1468.37**	*HGS*	0.89	0.65
*ATG12*	**3.31**	0.23	*HSP90AA1*	1.26	1.69
*ATG16L1*	**2.29**	0.78	*HSPA8*	**26579.01**	**7.89**
*ATG16L2*	0.00	0.71	*HTT*	**107.49**	0.78
*ATG3*	0.92	0.51	*IFNG*	1.26	1.69
*ATG4A*	1.04	**2.89**	*IGF1*	0.34	1.69
*ATG4B*	0.06	**4.11**	*INS*	**14.01**	**9.06**
*ATG4C*	**2.73**	0.68	*IRGM*	0.64	0.93
*ATG4D*	0.78	1.05	*LAMP1*	1.32	0.59
*ATG5*	**2.86**	1.19	*MAP1LC3A*	0.51	0.50
*ATG7*	0.79	0.42	*MAP1LC3B*	1.32	**4.11**
*ATG9A*	1.52	**2.89**	*MAPK14*	1.01	0.09
*ATG9B*	**2.69**	1.19	*MAPK8*	1.53	**2.64**
*BAD*	**11.22**	0.16	*MTOR*	1.73	1.82
*BAK1*	0.94	0.30	*NFKB1*	1.67	0.73
*BAX*	1.26	0.30	*NPC1*	0.90	1.27
*BCL2*	0.49	0.45	*PIK3C3*	1.39	**3.61**
*BCL2L1*	0.00	0.33	*PIK3CG*	0.91	1.22
*BECN1*	0.23	0.44	*PIK3R4*	**19.13**	1.29
*BID*	0.60	0.23	*PRKAA1*	0.68	0.66
*BNIP3*	1.26	1.69	*PTEN*	**9.37**	0.00
*CASP3*	**4.68**	**3.97**	*RAB24*	0.32	**5.03**
*CASP8*	0.82	1.05	*RB1*	**3.13**	1.04
*CDKN1B*	0.56	0.43	*RGS19*	0.89	0.29
*CDKN2A*	0.46	0.18	*RPS6KB1*	**4.05**	1.18
*CLN3*	0.83	0.45	*SNCA*	**7.77**	**6.19**
*CTSB*	0.08	0.00	*SQSTM1*	**18.10**	**9.00**
*CTSD*	**4.16**	0.88	*TGFB1*	1.89	0.18
*CTSS*	1.00	**4.56**	*TGM2*	0.83	0.32
*CXCR4*	1.08	**6.02**	*TMEM74*	**4.85**	0.92
*DAPK1*	0.41	0.61	*TNF*	1.28	4.06
*DRAM1*	1.76	1.84	*TNFSF10*	0.65	0.39
*DRAM2*	0.66	1.14	*TP53*	**16.66**	1.60
*EIF2AK3*	**8.16**	0.33	*ULK1*	1.31	1.41
*EIF4G1*	0.83	0.91	*ULK2*	**11.54**	**33.13**
*ESR1*	0.24	0.16	*UVRAG*	0.48	0.90
*FADD*	0.33	0.24	*WIPI1*	0.39	1.03
*FAS*	0.59	0.32	*ACTB*	1.55	**2.28**
*GAA*	**19.81**	**3.25**	*B2M*	1.55	0.54
*GABARAP*	0.68	**2.33**	*GAPDH*	0.26	0.60
*GABARAPL1*	0.29	0.99	*HPRT1*	0.44	0.32
			*RPLP0*	**3.63**	**4.23**

Fold-change values in the Met and 3-MA groups were calculated using the 2^–∆∆Ct^ formula and normalized to the values in the control group. Differences in expression more than twofold were accepted as the cut-off value (*n* = 3)

### Autophagy response modulation affected the expression of genes related to the wnt signaling pathway

To assess whether the inhibition/stimulation of autophagy can influence the Wnt signaling pathway, PCR array analysis was done (Table 2). Data indicated that the transcription of different genes related to several signaling transduction axes was altered in MSCs exposed to 15 µM Met and 3 µM 3-MA after 48 h. According to the data, treatment with Met can activate specific genes such as FZD9 (2.08-fold), CTNNB1 (2.02-fold), and SFRP4 (2.02-fold) belonging to the Canonical Wnt signaling pathway (Supplementary Table 2, Supplementary Fig. 1, and supplementary data File 1). Along with these changes, the expression of WNT2 (3.13.-fold), 7 A (3.68-fold), and 8 A (4.92-fold), SFRP1 (2.46-fold), NKD1 (2.41-fold), FZD8 (2.26-fold), DVL1 (3.83-fold), LRP5 (3.86-fold), DKK3 (2.03-fold), DIXDC1 (2.19-fold) from same signaling transduction pathway was increased significantly in 3-MA-treated group compared to the Met and control cells. Met had a potential to increase the expression of DAAM1 (2.29-fold) from the planar cell polarity signaling transduction pathway while 3-MA stimulated the transcription of NKD1, WNT2 (3.13.-fold), DVL1 (3.83-fold), VANGL2 (2.03-fold), WNT7A (3.68-fold), WNT8A (4.92-fold), and RHOU (2.25-fold) from the same molecular cascade. On the other hand, Wnt negative regulators such as KREMEN1 (3.83-fold), DKK3 (2.03-fold), NKD1, and SFRP1 (2.46-fold) were activated in the presence of 3 µM 3-MA. Our data also indicated that Met can activate SFRP4 (2.02-fold), CXXC4 (2.82-fold), and WNT16 (2.39-fold) from the same signaling axis compared to the control MSCs. Both Met and 3-MA triggered the FBXW11 (2.69-, and 4.62-fold), and TLE1 (2.71, and 5.65-fold), respectively from the same pathway. We found that Met can affect the MSC fate via the expression of CTNNB1 (2.02-fold). Interestingly, the transcription of VANGL2 belonging to tissue polarity was also increased in 3-MA-treated cells. Wnt signaling target genes such as DAB2, MYC, JUN, CCN4, and MMP7 were also altered in the presence of Met and 3-MA. Data indicated that the expression of genes belonging to cell growth and proliferation such as MYC (2.34-fold), JUN (2.6-fold), CTNNB1 (2.02-fold), DAB2 (2.21-fold), and MMP-7 (11.76-fold) was up-regulated in Met-treated MSCs compared to the non-treated control MSCs. Likewise, 3-MA induced the expression of CCN4 (2.82), LRP5, MMP7 (2.13-fold), and BCL9 (2.79-fold) in MSCs from the same signaling pathway. LRP5 can also control the cell migration capacity. We noted that Met can alter the expression of several genes related to cell cycle (MYC, JUN, and CTNNB1), homeostasis (MYC, and JUN), and activity signature genes (MYC) compared to the non-treated MSCs. 3-MA induced the expression of FBXW4 (2.23-fold) gene related to the Ubl conjugation pathway.

**Table 2:** PCR array analysis of human wnt signaling pathway in MSCs exposed to Met and 3-MA

Gene	Met	3-MA	Gene	Met	3-MA
*AES*	1.07	1.41	*LRP6*	0.79	1.13
*APC*	0.00	0.27	*MAPK8*	1.54	1.06
*AXIN1*	0.89	0.71	*MMP7*	**11.76**	**2.13**
*AXIN2*	0.80	1.84	*MYC*	**2.34**	0.48
*BCL9*	1.51	**2.79**	*NFATC1*	0.92	1.06
*BTRC*	1.08	1.34	*NKD1*	1.42	**2.41**
*CCND1*	1.11	1.92	*NLK*	1.37	1.27
*CCND2*	0.32	0.61	*PITX2*	1.80	1.44
*CSNK1A1*	1.95	1.20	*PORCN*	0.29	0.91
*CSNK2A1*	0.97	0.14	*PPARD*	0.70	1.52
*CTBP1*	0.22	0.91	*PRICKLE1*	0.50	0.56
*CTNNB1*	**2.02**	1.57	*PYGO1*	1.44	1.16
*CTNNBIP1*	0.71	1.78	*RHOA*	1.05	0.15
*CXXC4*	**2.82**	1.16	*RHOU*	1.44	**2.25**
*DAAM1*	**2.29**	0.80	*RUVBL1*	0.80	1.11
*DAB2*	**2.21**	1.70	*SFRP1*	1.42	**2.46**
*DIXDC1*	1.76	**2.19**	*SFRP4*	**2.02**	1.73
*DKK1*	0.45	0.82	*SOX17*	0.07	0.50
*DKK3*	1.87	**2.03**	*TCF7*	0.50	0.91
*DVL1*	1.09	**3.83**	*TCF7L1*	0.07	0.49
*DVL2*	0.49	0.90	*TLE1*	**2.71**	**5.65**
*EP300*	0.70	1.44	*VANGL2*	1.36	**2.03**
*FBXW11*	**2.69**	**4.62**	*WIF1*	0.10	0.21
*FBXW4*	1.32	**2.23**	*CCN4*	1.11	**2.82**
*FGF4*	0.42	0.75	*WNT1*	1.07	1.60
*FOSL1*	1.02	0.86	*WNT10A*	1.33	1.88
*FOXN1*	0.82	1.17	*WNT11*	1.00	1.24
*FRAT1*	0.69	0.59	*WNT16*	**2.39**	1.79
*FRZB*	1.68	1.24	*WNT2*	1.81	**3.13**
*FZD1*	1.20	1.22	*WNT2B*	0.00	0.07
*FZD2*	1.48	1.18	*WNT3*	1.63	1.02
*FZD3*	1.38	1.36	*WNT3A*	1.07	1.42
*FZD4*	1.51	1.56	*WNT4*	0.47	1.43
*FZD5*	1.29	1.04	*WNT5A*	1.22	1.44
*FZD6*	0.97	0.96	*WNT5B*	0.24	0.62
*FZD7*	1.68	1.79	*WNT6*	0.99	1.34
*FZD8*	0.97	**2.26**	*WNT7A*	1.44	**3.68**
*FZD9*	**2.08**	1.69	*WNT7B*	0.05	0.34
*GSK3A*	0.87	0.94	*WNT8A*	1.63	**4.92**
*GSK3B*	0.91	0.28	*WNT9A*	1.54	1.17
*JUN*	**2.60**	1.44	*ACTB*	1.11	1.93
*KREMEN1*	0.84	**3.83**	*B2M*	0.94	1.37
*LEF1*	1.80	1.81	*GAPDH*	0.82	1.38
*LRP5*	1.62	**3.86**	*HPRT1*	1.63	1.20
			*RPLP0*	0.71	0.23

Fold-change values in the Met and 3-MA groups were calculated using the 2^–∆∆Ct^ formula and normalized to the values in the control group. Differences in expression more than twofold were accepted as the cut-off value (*n* = 3)

### Protein array analysis

**Fig. 5:**
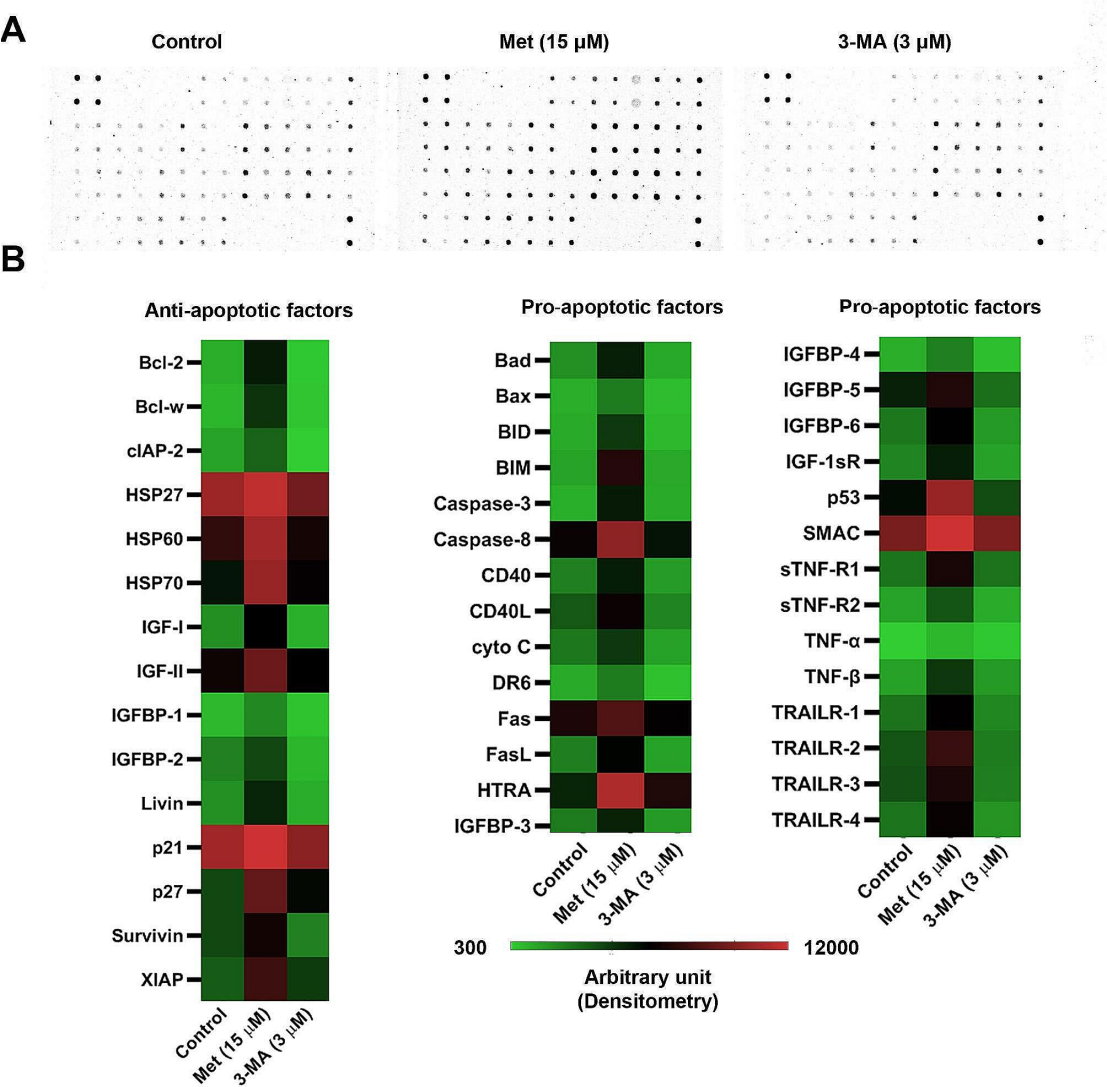
Measuring protein levels of 43 factors (pro- and anti-apoptotic proteins) by human apoptosis antibody array in MSCs exposed to 15 µM Met, and 3 µM 3-MA after 48 h. Data were obtained from three pooled samples

The protein levels of pro- and anti-apoptotic factors were measured using a human apoptosis antibody array in MSCs after being exposed to Met and 3-MA (Fig. 5). Based on the data, incubation of MSCs with Met increased protein levels of anti-apoptosis factors such as Bcl-2 (3.37-fold), Bcl-w (3.41-fold), IGF-I (2.53-fold), Livin (2.17-fold), p27 (2.04-fold), and XIAP (2.05-fold) compared to the non-treated control MSCs (Supplementary Table 3, supplementary Fig. 2, and Supplementary Data File 2). Compared to non-treated control MSCs, pro-apoptotic factors, including Bad (2.63-fold), Bax (2.27-fold), BID (3.67-fold), BIM (4.85-fold), Caspase-3 (4.77-fold), CD40 (2.15-fold), DR6 (2.07-fold), FasL (2.41-fold), HTRA (2.16-fold), IGFBP-4 (2.14-fold), IGFBP-6 (2.25-fold), IGF1-sR (2.26-fold), sTNF-R1 (2.44-fold), sTNF-R2 (2.5-fold), TNF-α (3.23-fold), TNF-β (3-fold), TRAILR-1 (2.18-fold), TRAILR-2 (2.08-fold), and TRAILR-4 (2.25-fold) were also elevated in the presence of Met. Data showed the lack of changes in these proteins in 3-MA-treated MSCs. These data showed that Met, but not 3-MA, altered protein levels of the apoptosis signaling pathway, either pro-apoptotic or anti-apoptotic factors.

### Fatty acid profile was changed after the modulation of autophagy in MSCs

**Fig. 6:**
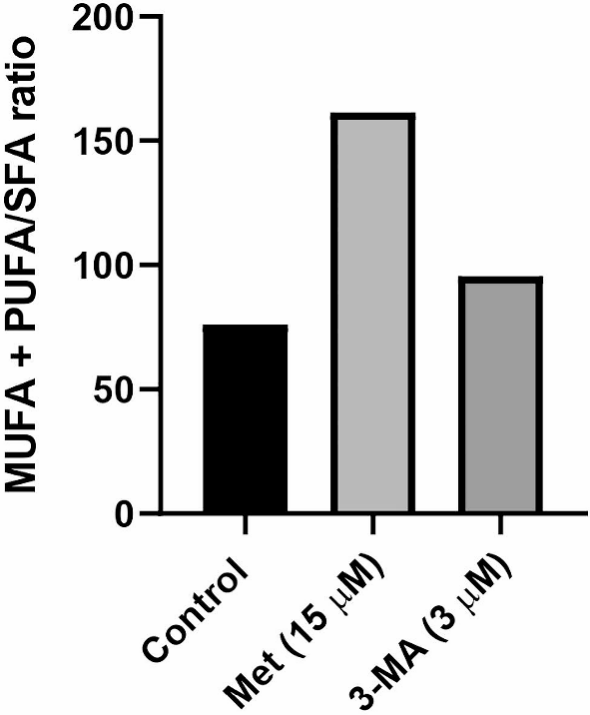
Fatty acid profile analysis using gas chromatography. Data indicated the increase of PUFA + MUFA/SFA ratio in Met-treated MSCs compared to control and 3-MA groups. Data were obtained from three pooled samples

In the current experiment, the fatty acid profile was studied using GS. The level of PUFA (Linoleate18:20, MUFA (Oleate 18:1), and SFA (Myristate14:0, Palmitate 16:0, Stearate 18:0 and Pentadecanoic acid 15:0) was measured in the presence of Met and 3-MA (Fig. 6). Data showed the increase of PUFA + MUFA/SFA ratio in Met-treated MSCs (161.45) compared to control and 3-MA treated MSCs. The PUFA + MUFA/SFA ratios were 76.12, and 95.51, respectively in the control and 3-MA-treated MSCs. These data indicate that autophagy modulators can alter the MSC fatty acid profile.

## Discussion

Emerging data have indicated the critical role of autophagy in various cell activities. Whether and how autophagy modulation can affect the homotypic juxtracrine interaction between the MSCs is the subject of debate. Here, we investigated the effect of autophagy modulation using Met and 3-MA on intercellular communication via TNT formation. The impact of autophagic response was also investigated in terms of mitochondrial donation and internalization. Incubation of human MSCs with different doses of Met, 1, 5, 15, 20, 50, and 75 µM, induced survival rate. It was suggested that Met can postpone the aging of MSCs in a Nrf2/GPx7-dependent manner [26]. Based on the previous findings, an increased autophagy response can preserve the self-renewal and multigenerational differentiation capacity of MSCs and postpone aging-related changes after long-term culture in vitro [27, 28]. It has been shown that Met can reduce the survival rate of MSCs in a dose-, and time-dependent manner [29]. It seems that the higher doses of Met reduce the proliferation rate of MSCs while enhancing differentiation capacity into several lineages [30, 31]. According to different studies, Met can affect the viability of various cell types via the modulation of several intracellular factors. Protein array analysis indicated that Met can alter protein levels of both pro- and anti-apoptotic factors inside the MSCs after 48 h. The increase of p21 in Met-treated MSCs is associated with the inhibition of apoptosis and DNA repair [32]. Besides, the increase of HSP27, HSP60, and HSP70 in the presence of Met showed the reduction of endoplasmic reticulum stress [33]. Factors such as IGFII, and XIAP, a Caspase inhibitor, are associated with the inhibition of apoptosis in MSCs [34, 35]. While several pro-apoptotic factors such as p27, Caspase 8, and HTRA were simultaneously increased. According to the MTT data panel, it seems that the increase of multiple pro-apoptotic mediators is a compensatory response to Met treatment and does not affect the survival rate. In line with this statement, incubation of MSCs with Met can activate shared autophagy and apoptosis mediators such as ATG12, 5, BAD, EIF2AK3, HDAC1, HTT, PTEN, SNCA, SQSTM1, and TP53. Thus, one can hypothesize that the activation of autophagy/apoptosis is an essential phenomenon to increase the therapeutic effects of stem cells [36].

To modulate the autophagy response, MSCs were treated with 15 µM Met and 3 µM 3-MA for 48 h. Western blotting revealed the lack of statistically significant differences in protein levels of BCLN1, p62, and LC3-II/LC3-I ratio in Met- and 3-MA-treated cells compared to the control group. On the contrary, IF staining confirmed the increase in Green LysoTracker acidic compartments and autophagic flux in Met groups compared to non-treated MSCs. These features were blunt in the 3-MA group as compared to control MSCs. The increase of acidic compartments indicates the formation of autophagolysosomes and promotes autophagy response [37]. Along with these data, the expression of multiple autophagy stimulators such as AT10, 12, 16L1, 4 C, 5, ULK1, and 9B was upregulated in Met-treated MSCs, indicating the promotion of vesicle formation. Data also confirmed the activation of both stimulatory and inhibitory autophagy mediators in the presence of 3 µM 3-MA. Highly expressed HSPA8 showed the activation of CMA in the presence of Met. The data showed that incubation of MSCs with Met or 3-MA can modulate multiple mediators related to the autophagy signaling pathway.

To show the possible impact of autophagy modulation on TNT formation, bright-field, and SEM images were taken after 48 h. We also found that 15 µM Met had the potential to stimulate TNT formation and cell projection (lamellipodia, and filopodia). Based on the obtained data, the number and length of connecting TNTs were increased in MSCs in the presence of Met while opposite effects were found in 3-MA-treated group. It has been shown that cytoskeletal remodeling is integral to the formation of TNTs and membrane protrusions [38, 39]. One reason for the increase of TNT, lamellipodia, and filopodia is that Met can increase F-actin assembly via the increase of Cdc42 expression. This feature can affect the regenerative potential of neural progenitor cells by influencing the migration capacity and differentiation into the target lineages under ischemic conditions [40]. Śmieszek et al. declared that treatment of MSCs with Met led to the formation of cell projection in a dose-dependent manner in which 1 mM Met induced cell lamellipodia, and filopodia rather than higher doses (5, and 10 mM) with irregularly shaped projections [31]. Western blotting indicated the reduction of TNT assembly system mediators (p-FAK and Rab8) in the 3-MA group compared to control MSCs. It has been shown that the activation of the Rab8a/Rab11/VAMP3 axis is associated with the formation of TNTs but not filopodia. Thus, it should be noted that different stimulators are mandatory to promote the formation of TNTs, and filopodia [41]. Even though, the existence of varied TNT structures with different lengths and thicknesses may pre-determine the formation of certain TNT types under specific treatment conditions [42]. In contrast to our finding, Hakimee and co-workers found that higher Met concentrations (0–10 mM) can reduce HeLa cell migration via the reduction of p-FAK/FAK ratio, Rac1 and RhoA, and thus lamellipodia and filopodia formation [43]. In a similar study, incubation of mesothelioma cells with different concentrations of Everolimus (20, 40, and 80 µM), an mTOR inhibitor, reduced TNT and cell projections over time [44]. Long-period incubation time may reverse the formation of TNTs in human MSCs. Enhanced green MitoTracker^+^ particles in Met-treated cells indicated appropriate mitochondrial membrane integrity and number compared to the other groups [45]. Along with data, the transfer of cargo via TNTs was evident between the MSCs after exposure to Met. Despite these data, we did not find any significant changes in protein levels of Miro-1 and − 2 using western blotting. Miro-1 and − 2 can directly affect the subcellular localization of mitochondria via microtubule- and actin-based mechanisms [46]. Miro-1 and − 2 have GTPase activity and act as connectors for mitochondrial membranes and motor proteins (i.e. Dynein, Kinesin-1) and adaptor proteins (TRAK1/2) [47]. One possible reason for the lack of significant difference in protein levels of Miro-1, and − 2 in the presence of Met would be that MSCs have higher mitochondrial transport activity compared to other cells [48]. Thus, one can hypothesize that 15 µM Met used in this study for 48 h did not provoke additional Miro-1, and − 2 synthesis and activity. Of course, RT-PCR array analysis showed the expression of several mediators associated with vesicle transport, indicating the stimulation of other factors involved in organelle sublocalization. Previous data confirmed the close relationship between the canonical autophagy machinery and TNT apparatus. For example, de Rooij et al., indicated that LC3B^+^ autophagosomes and mitochondrial mass are present in the TNT lumen and transported between cells [49]. On the other hand, the intracellular compartment between the cells needs motor mediators such as Miro1, and cytoskeletal filaments [50]. In response to the activation of autophagy, the phosphorylated Miro1 can transfer injured mitochondria with the collaboration of certain motor proteins [51]. It seems that under stressful conditions, the activity of specific protein complex such as TNFAIP2/M-Sec can also promote TNTs after autophagic response under diabetic conditions. To be specific, the elevation of p62, and LC3 can foster the formation of TNT for the elimination of injured mitochondria via TNT compartment [52].

We also found that the inhibition of autophagy slightly increased the internalization rate of free mitochondrial particles (43.6 ± 10.5 vs. 33.6 ± 1.1%) compared to the control MSCs. However, these values did not reach statistically significant levels. Despite the non-significant results, it should be said that trivial changes in fluorescence intensity as shown by flow cytometry can encompass several mitochondrial particles that are internalized in response to the inhibition of autophagy response. It has been shown that the inhibition of autophagy can increase mitochondrial DNA injury via the modulation of fission, biogenesis, and mitophagy, leading to the reduction of active mitochondrial particles and a metabolism switch from the oxidation-phosphorylation toward glycolysis [53]. On the other hand, the inhibition of mitochondrial function, or depletion of mitochondrial DNA increases the possibility of mitochondria internalization in homotypic and heterotypic cells via micropinocytosis [54]. Thus, it is logical to hypothesize that these conditions make the host cell eligible to accept the external mitochondria from the juxtaposed cells.

The changes in MUFA, PUFA, and SFA profiles were monitored after autophagy modulation. Previous data have confirmed the close relationship between the lipid metabolism and autophagy response [55, 56]. The current study indicated the increase of MUFA + PUFA/SFA ratio in Met-treated MSCs after 48 h. In terms of TNT, and cell projection formation, the increase of unsaturated fatty acids can promote cell membrane fluidity in which higher cholesterol and SFA lead to the acquisition of more rigid membrane features [57]. Of note, the changes in lipid content by an autophagy inhibitor, chloroquine, can accumulate intracellular lipids and thus inhibit autophagy flux [58]. It has been shown that the increase of unsaturated fatty acids influences the fluidity, curvature, and stiffness of cell membrane via providing single or several bents at the site of double bonds, leading to poorly organized through the bilipid layer. These features potentiate the host cells to provide several types of projections to the surrounding milieu [59]. Turner et al. previously declared that the generation of TNT-like protrusions and elongated morphologies is closely associated with PUFA metabolism [60]. Of course, it should not be forgotten that there is a close relationship between autophagy status and unsaturated fatty acids [61]. It was suggested that dietary ω-6 PUFA stimulates the autophagic flux in mTOR-dependent and mTOR-independent manner [62]. Like to present study, we previously indicated that treatment of human stem cells with Met can increase the PUFA and MUFA contents [61].

We also found that the treatment of stem cells with autophagy modulators can affect the mediators of the Wnt signaling pathway. The critical role of the Wnt/PCP pathway has been indicated in stem cell cytoskeletal remodeling, morphogenesis, and polarity via the regulation of c-JNK, Cdc42, and RhoA [63]. As such, our data indicated the expression of these genes in Met-treated MSCs. Along with these statements, De Calisto et al. demonstrated that cytoskeletal behavior and cell adhesion properties are controlled by Wnt in the neural crest. The activity of the Wnt signaling pathway is required for the generation of the cell protrusions. They found that the Wnt/planar cell polarity (PCP) signaling pathway precisely controls the formation of lamellipodia and filopodia in the neural crest cells [64]. Notably, the inhibition of Wnt signaling pathway mediators such as Vangl2, or interruption of JNK can shorten the generated filopodia [65]. The control of Wnt/Ca^2+^ can affect the dynamic morphogenesis of TNTs and TNT-mediated vesicle transfer via the regulation of /calmodulin-dependent protein kinase II (CaMKII) in CAD cells, indicating the critical role of the Wnt signaling pathway in TNT propagation [66].

## Conclusion

The regulation of autophagy can influence several MSC bioactivates mainly juxtacrine interaction with the other cells. The formation of TNTs in MSCs can be associated with autophagy modulation via different underlying mechanisms.

### Electronic supplementary material

Below is the link to the electronic supplementary material.

Supplementary Material 1

Supplementary Material 2

Supplementary Material 3

Supplementary Material 4

Supplementary Material 5

Supplementary Material 6

Supplementary Material 7

Supplementary Material 8

Supplementary Material 9

Supplementary Material 10

Supplementary Material 11

Supplementary Material 12

Supplementary Material 13

Supplementary Material 14

## Data Availability

The authors confirm that the data supporting the findings of this study are available within the article and supplementary materials.

## Abbreviations

3-MA: 3-Methyladenine
ATGs: Autophagy-related genes
BECLN-1: Beclin-1
CaMKII: Calmodulin-dependent protein kinase II
DMSO: Dimethyl sulfoxide
EVs: Extracellular vesicles
FAK: Focal adhesion kinase
HRP: Horseradish peroxidase
FBS: Fetal bovine serum
DMEM/LG: Low-glucose content Dulbecco’s modified Eagle’s Medium
MSCs: Mesenchymal stem cells
Met: Metformin
Miro1 and 2: Mitochondrial Rho-GTPases 1 and 2
MUFAs: Monounsaturated fatty acids
PCP: Planar cell polarity
PUFAs: Polyunsaturated fatty acids
SFA: Saturated fatty acids
SEM: Scanning electron microscopy
TNTs: Tunneling nanotubes
