# Supplementary figures and images for: Autophagy modulation effect on homotypic transfer of intracellular components via tunneling nanotubes in mesenchymal stem cells
Source: Stem Cell Res Ther. 2024 Jul 2;15:189. doi: 10.1186/s13287-024-03813-1 (PMC11218273; doi:10.1186/s13287-024-03813-1)

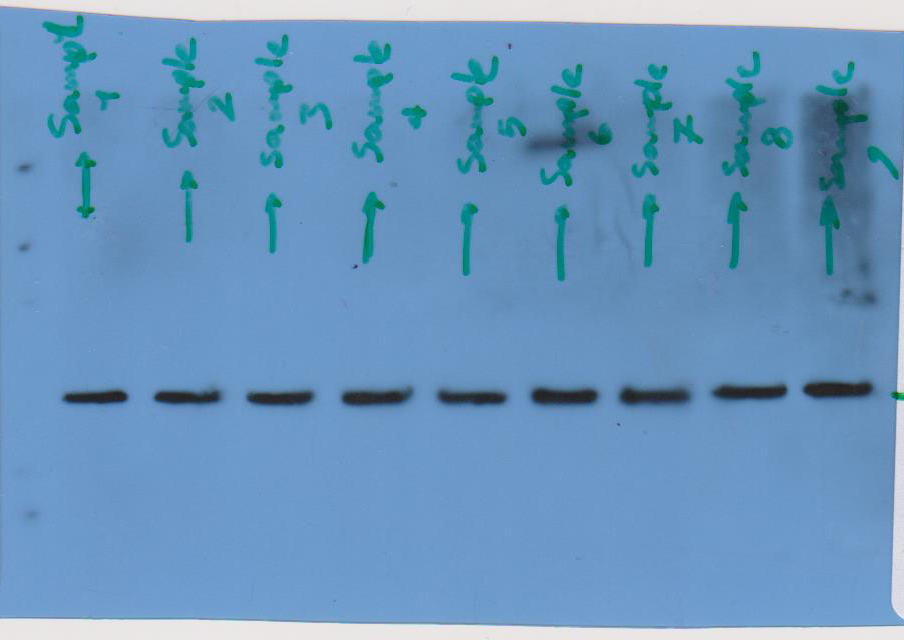

Supplement: Supplementary file 1 — Supplementary Material 1 [file 13287_2024_3813_MOESM1_ESM.jpg]

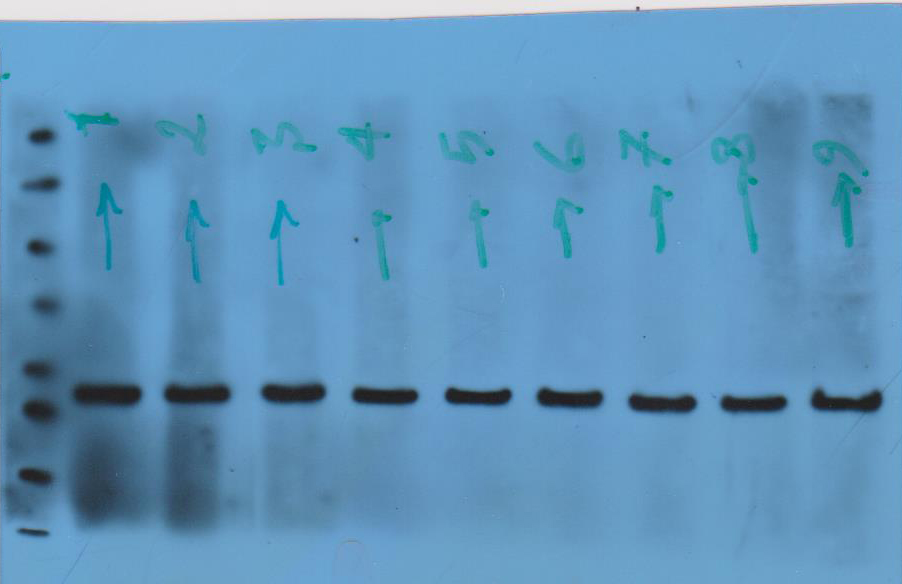

Supplement: Supplementary file 2 — Supplementary Material 2 [file 13287_2024_3813_MOESM2_ESM.jpg]

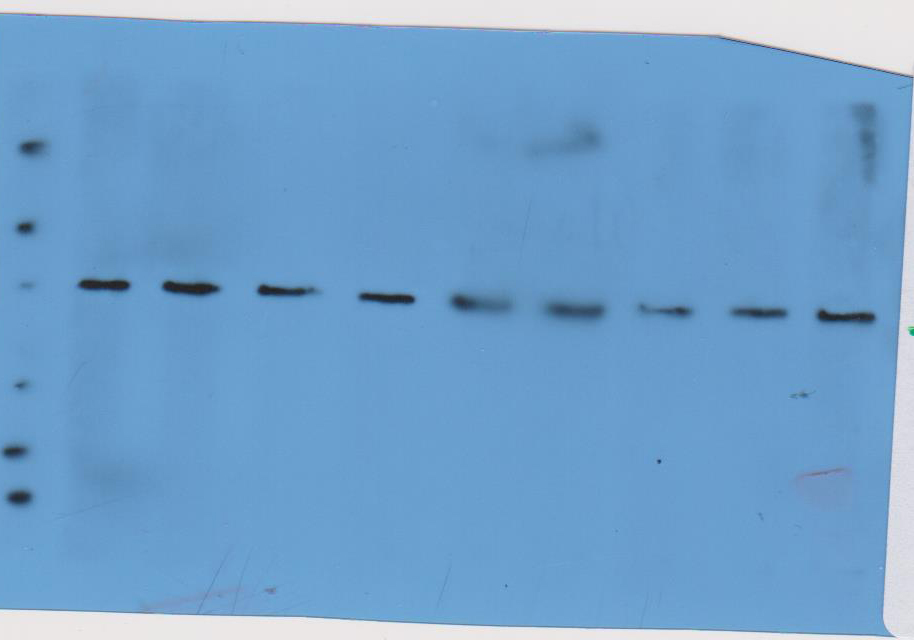

Supplement: Supplementary file 3 — Supplementary Material 3 [file 13287_2024_3813_MOESM3_ESM.jpg]

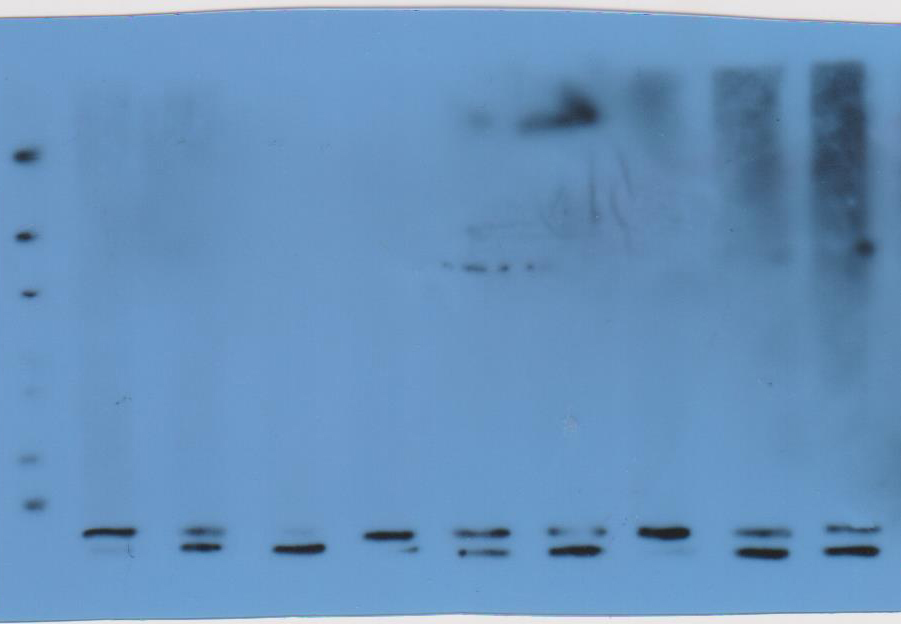

Supplement: Supplementary file 4 — Supplementary Material 4 [file 13287_2024_3813_MOESM4_ESM.jpg]

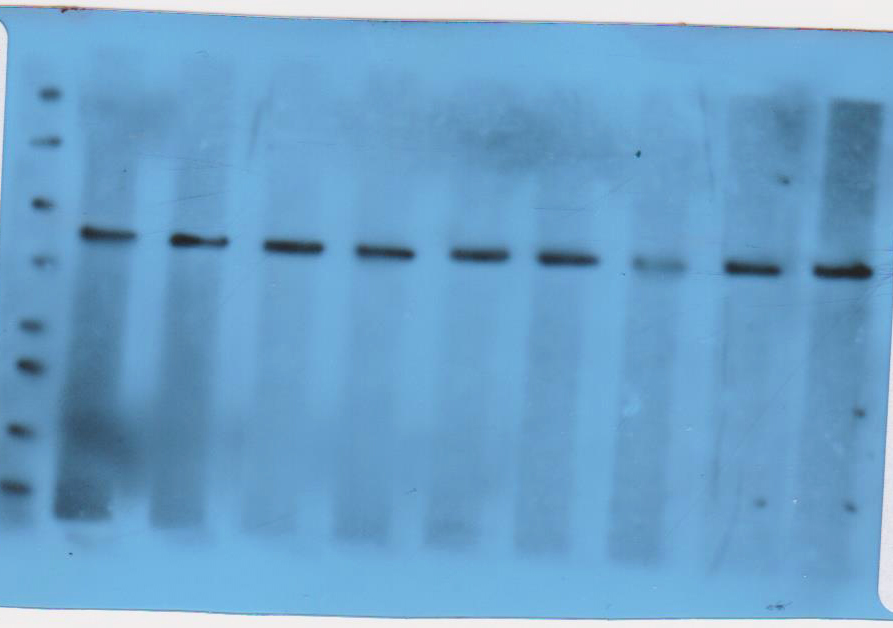

Supplement: Supplementary file 5 — Supplementary Material 5 [file 13287_2024_3813_MOESM5_ESM.jpg]

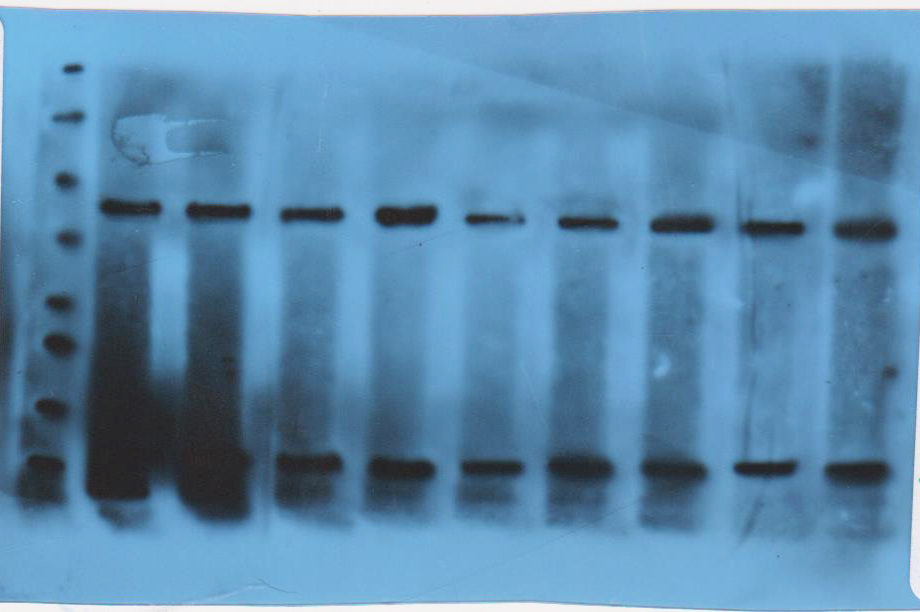

Supplement: Supplementary file 6 — Supplementary Material 6 [file 13287_2024_3813_MOESM6_ESM.jpg]

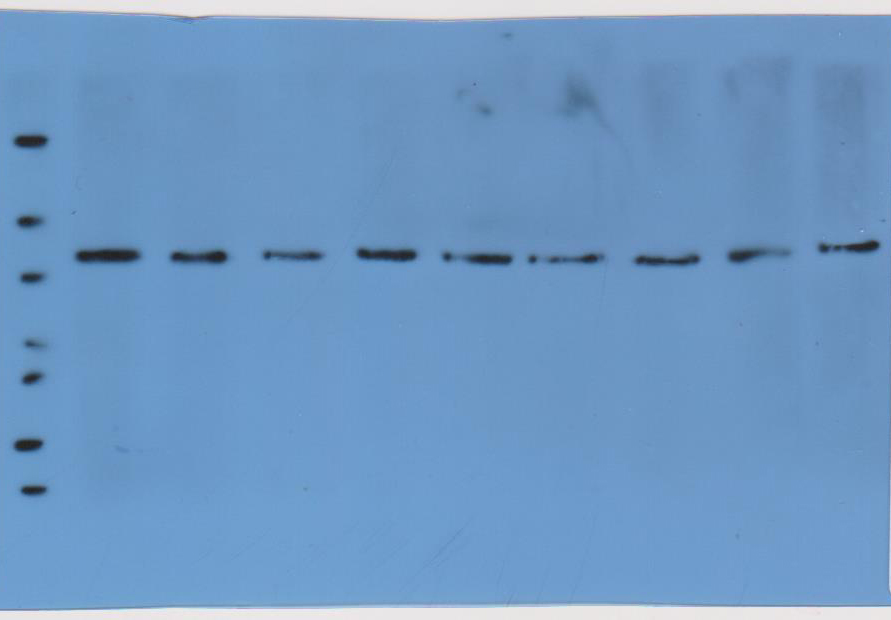

Supplement: Supplementary file 7 — Supplementary Material 7 [file 13287_2024_3813_MOESM7_ESM.jpg]

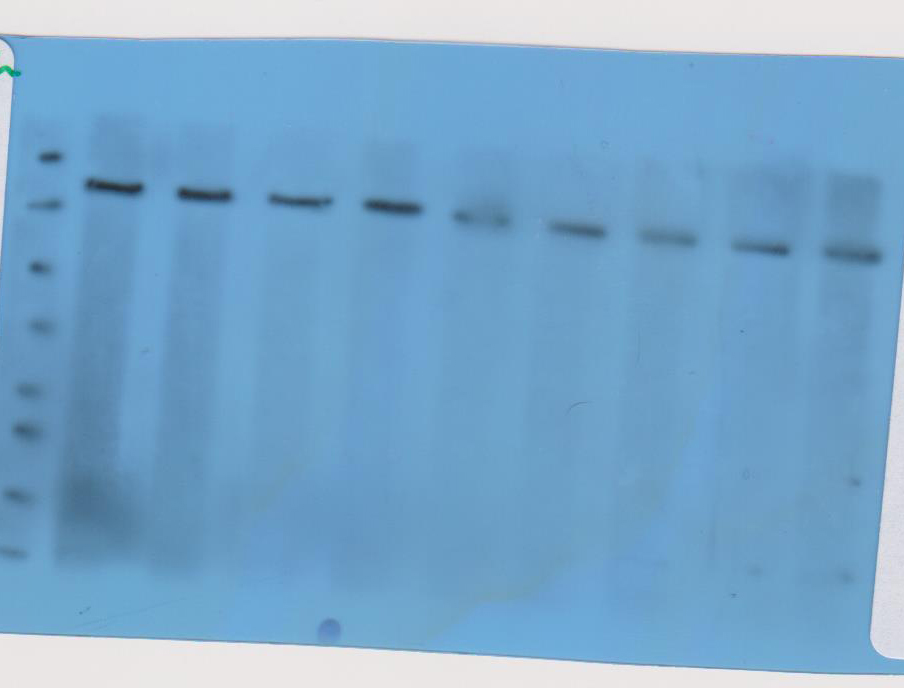

Supplement: Supplementary file 8 — Supplementary Material 8 [file 13287_2024_3813_MOESM8_ESM.jpg]

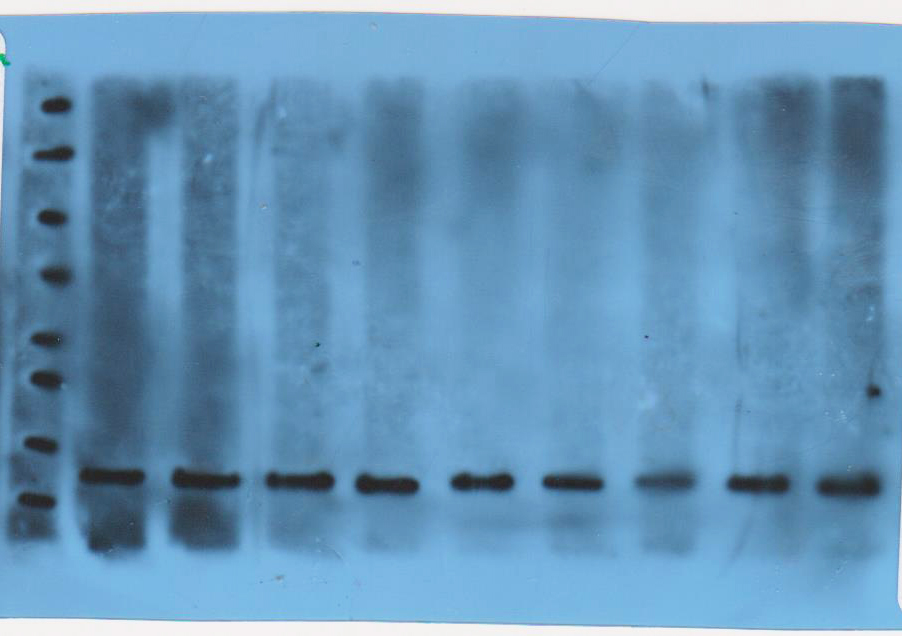

Supplement: Supplementary file 9 — Supplementary Material 9 [file 13287_2024_3813_MOESM9_ESM.jpg]

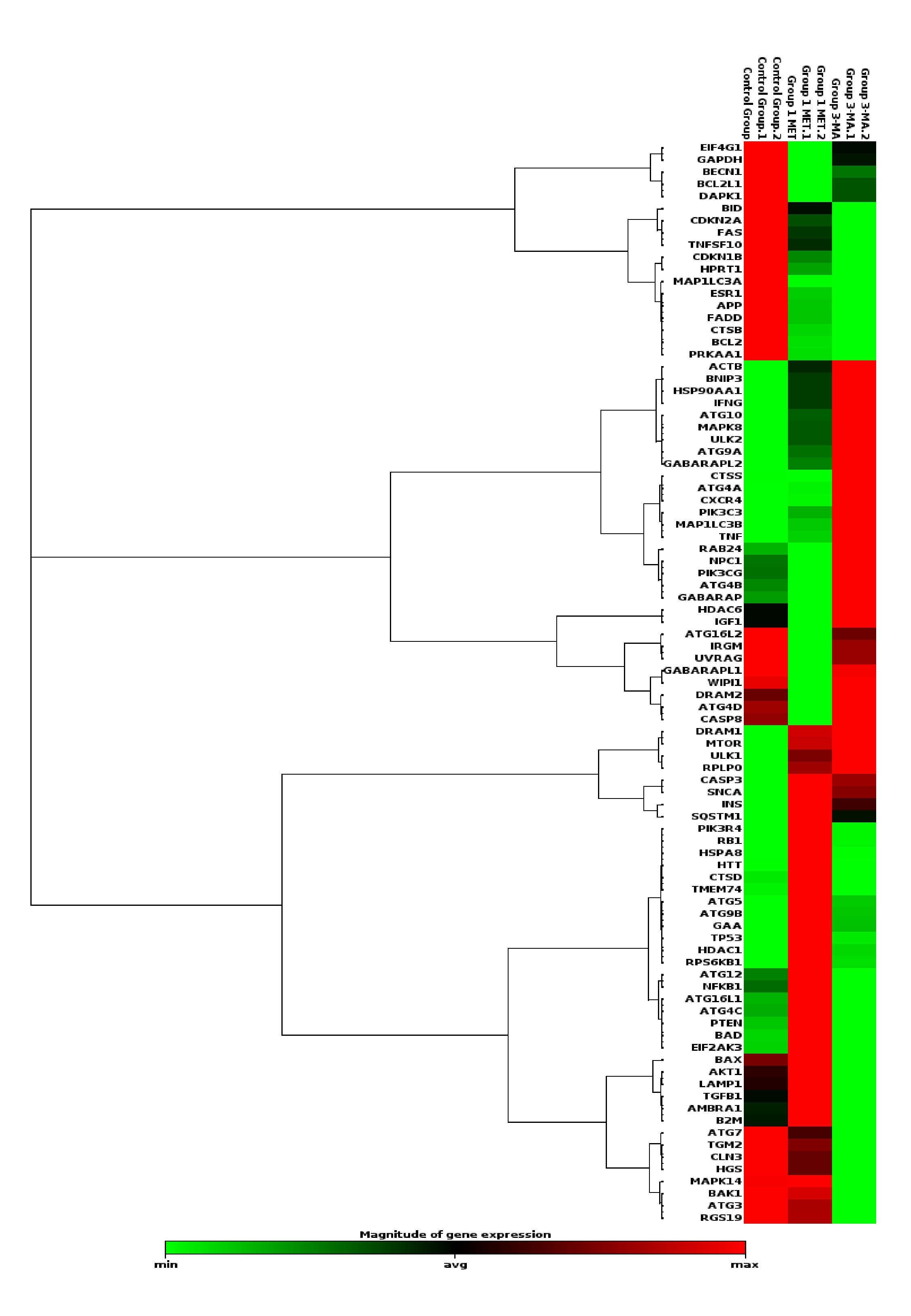

Supplement: Supplementary file 10 — Supplementary Material 10 [file 13287_2024_3813_MOESM10_ESM.jpg]

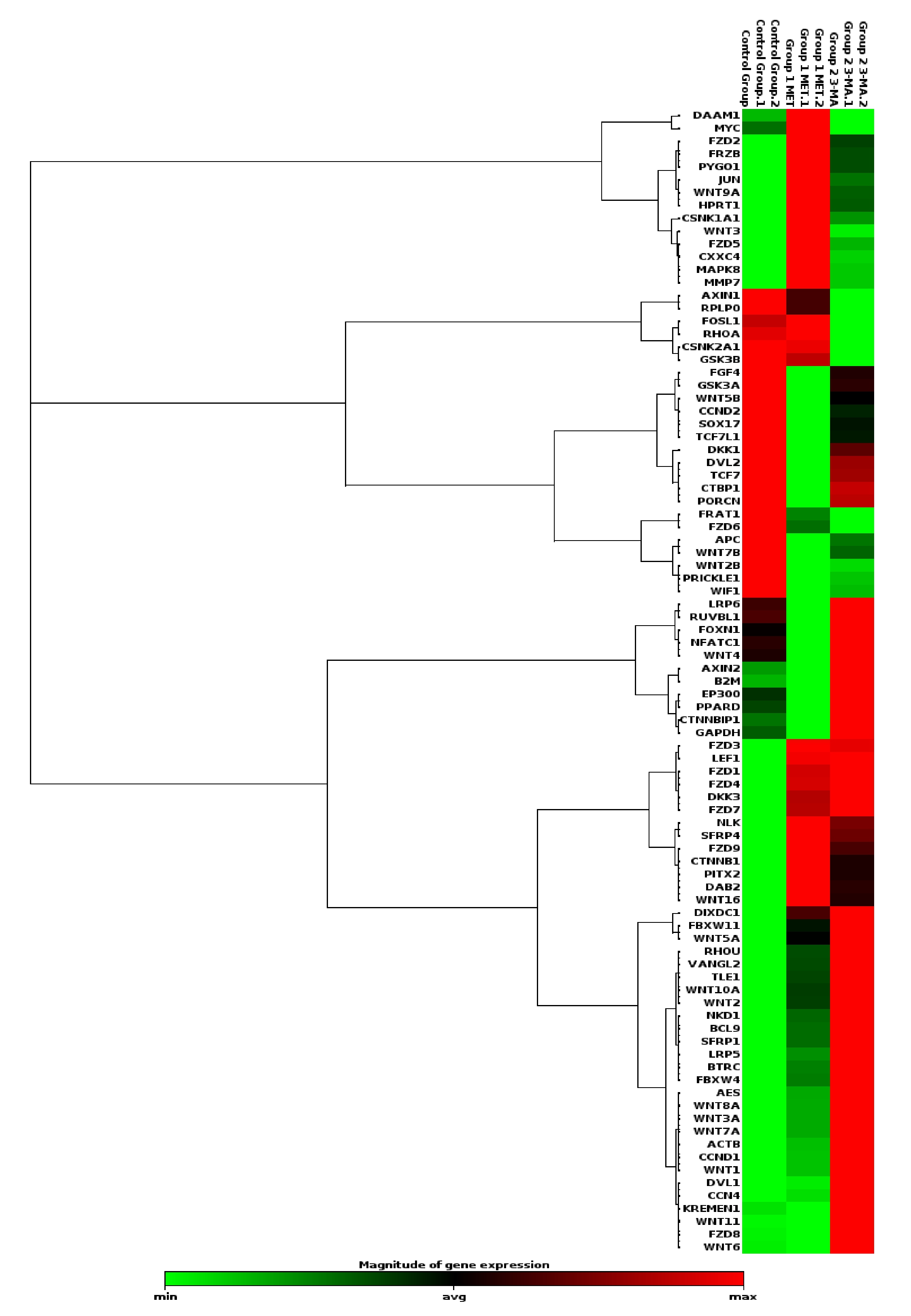

Supplement: Supplementary file 11 — Supplementary Material 11 [file 13287_2024_3813_MOESM11_ESM.jpg]
