# Supplementary material for: Autophagy modulation effect on homotypic transfer of intracellular components via tunneling nanotubes in mesenchymal stem cells
Source: Stem Cell Res Ther. 2024 Jul 2;15:189. doi: 10.1186/s13287-024-03813-1 (PMC11218273; doi:10.1186/s13287-024-03813-1)
Supplement: Supplementary file 12 — Supplementary Material 12 [file 13287_2024_3813_MOESM12_ESM.docx]

**Supplementary Table 1**. Different Autophagy signal transduction pathways

| Different autophagy signal transduction pathways | List of genes |
| --- | --- |
| Autophagic Vacuole Formation | AMBRA1 (NYW1), ATG12, ATG16L1, ATG4A, ATG4B, ATG4C, ATG4D, ATG5, ATG9A, ATG9B, BECN1, GABARAP, GABARAPL1, GABARAPL2, IRGM, MAP1LC3A, MAP1LC3B, RGS19, ULK1, WIPI1 |
| Vacuole Targeting | ATG4A, ATG4B, ATG4C, ATG4D, GABARAP |
| Protein Transport | ATG10, ATG16L1, ATG16L2, ATG3, ATG4A, ATG4B, ATG4C, ATG4D, ATG7, ATG9A, GABARAP, GABARAPL2, RAB24 |
| Autophagosome-Lysosome Linkage | DRAM1, GABARAP, LAMP1, NPC1 |
| Ubiquitination | ATG3, ATG7, HDAC6 |
| Proteases | ATG4A, ATG4B, ATG4C, ATG4D |
| Co-Regulators of Autophagy & Apoptosis | AKT1, APP, ATG12, ATG5, BAD, BAK1, BAX, BCL2, BCL2L1 (BCLXL), BECN1, BID, BNIP3, CASP3, CASP8 (FLICE), CDKN1B (P27KIP1), CDKN2A (p16INK4a), CLN3, CTSB, CXCR4, DAPK1, DRAM1, EIF2AK3, FADD, FAS (TNFRSF6), HDAC1, HTT, IFNG, IGF1, INS, MAPK8 (JNK1), MTOR, NFKB1, PIK3CG, PRKAA1(AMPK), PTEN, SNCA, SQSTM1, TGFB1, TGM2, TNF, TNFSF10 (TRAIL), TP53 (p53) |
| Co-Regulators of Autophagy & the Cell Cycle | BAX, CDKN1B (P27KIP1), CDKN2A (p16INK4a), IFNG, PTEN, RB1, TGFB1, TP53 (p53) |
| Autophagy Induction by Intracellular Pathogens | EIF2AK3, IFNG, LAMP1 |
| Autophagy in Response to Other Intracellular Signals | CTSD, CTSS, DRAM2 (TMEM77), EIF4G1, ESR1 (ERα), GAA, HGS, MAPK14 (p38ALPHA), PIK3C3 (Vps34), PIK3R4, RPS6KB1, TMEM74, ULK2, UVRAG |
| Chaperone-Mediated Autophagy | HSP90AA1, HSPA8 |

**Supplementary Table 2**. Different Wnt signal transduction pathways

| Different Wnt signal transduction pathways | List of genes |
| --- | --- |
| Canonical WNT Signaling | APC, AXIN1, AXIN2, CSNK1A1, CTBP1, CTNNB1, CTNNBIP1 (ICAT), DKK1,DKK3, DVL1, DVL2, EP300, FRAT1, FZD1, FZD2, FZD3, FZD4, FZD5, FZD6, FZD7, FZD8, FZD9, GSK3B, LEF1, LRP5, LRP6, NKD1, PORCN, RUVBL1, SFRP1, SFRP4, SKP2, SOX17, TCF7, TCF7L1, WIF1, WNT1, WNT10A, WNT2, WNT2B, WNT3, WNT3A, WNT4, WNT6, WNT7A, WNT7B, WNT8A and DIXDC1 |
| Planar Cell Polarity (PCP) | DAAM1, DVL1, DVL2, MAPK8 (JNK1), NKD1, PRICKLE1, RHOA, VANGL2, WNT9A.  WNT/Calcium Signaling FZD2, NFATC1, WNT1, WNT10A, WNT11,WNT2, WNT2B, WNT3, WNT3A, WNT4, WNT5A, WNT5B, WNT6, WNT7A, WNT7B, WNT8A, WNT9A and RHOU |
| WNT Signaling Negative Regulation | APC, AXIN1, AXIN2, BTRC (bTrCP), CCND1, CTBP1, CTNNBIP1 (ICAT), DKK1, DKK3, FBXW11, FRZB (FRP-3), KREMEN1, LRP6, NKD1, NLK,SFRP1, SFRP4, SOX17, WIF1 and CXXC4, TLE1, WNT16 |
| WNT Signaling Target Genes | AXIN2, BTRC (bTrCP), CCN4, CCND1, CCND2, DAB2, FOSL1 (FRA-1), JUN, MMP7, MYC, PITX2, and PPARD |
| Cell Fate | CTNNB1, DKK1, WNT1, WNT3, and WNT3A |
| Tissue Polarity | AXIN2, FZD2, FZD3, FZD5, FZD6, and VANGL2 |
| Cell Growth & Proliferation | APC, CCN4, CCND1, CCND2, CTBP1, CTNNB1, CTNNBIP1 (ICAT), DAB2, EP300, FGF4, FOSL1 (FRA-1), FZD3, JUN, LRP5, MMP7, MYC, PPARD, *BCL9* and WNT3A |
| Cell Migration | APC, DKK1, LRP5, LRP6, RHOA, and WNT1 |
| Cell Cycle | APC, BTRC (bTrCP), CCND1, CCND2, CTNNB1, EP300, FOSL1 (FRA-1), JUN, MYC, RHOA, RUVBL1, and TCF7L1 |
| Cellular Homeostasis | APC, FZD2, JUN, and MYC |
| Pathway Activity Signature Genes | BOD1, CALM1, CCND1, CCND2, CHSY1, CXADR, CYP4V2, HSPA12A, LEF1, MT1A, MTFP1, MTSS1, MYC, NAV2, PRMT6, and SKP2 |
| Ubl Conjugation Pathway | FBXW4 |

**Supplementary Table 3**. Monitoring apoptosis-related proteins in human MSCs treated with Met and 3-MA.

| Genes | Control | Met | 3-MA |  | Met | 3-MA |
| --- | --- | --- | --- | --- | --- | --- |
|  | **Arbitrary units** | | |  | **Fold changes compared to control group** | |
| Bad | 2013.635 | 5300.206 | 1312.715 |  | 2.632158261 | 0.651913182 |
| Bax | 1155.284 | 2624.458 | 723.6081 |  | 2.271699426 | 0.626346509 |
| Bcl-2 | 1715.697 | 5790.961 | 1005.436 |  | 3.375281883 | 0.586021891 |
| Bcl-w | 1496.568 | 5106.77 | 1083.493 |  | 3.412320723 | 0.723985145 |
| BID | 1230.3 | 4515.983 | 844.8664 |  | 3.670635617 | 0.68671576 |
| BIM | 1475.631 | 7168.023 | 1336.024 |  | 4.857598546 | 0.90539166 |
| Caspase 3 | 1135.176 | 5415.915 | 1284.954 |  | 4.770991459 | 1.131942536 |
| Caspase 8 | 6400.468 | 10196.18 | 5599.204 |  | 1.593036634 | 0.874811654 |
| CD40 | 2479.337 | 5343.475 | 1693.191 |  | 2.155203185 | 0.682920878 |
| CD40L | 3619.285 | 6429 | 2321.005 |  | 1.776317698 | 0.641288265 |
| c IAP-2 | 2018.929 | 3793.073 | 874.6257 |  | 1.878755023 | 0.433212708 |
| cyto C | 2717.779 | 4553.175 | 1524.005 |  | 1.675329377 | 0.560753836 |
| DR 6 | 1253.739 | 2603.377 | 591.8648 |  | 2.076490402 | 0.472079755 |
| Fas | 6941.906 | 8437.483 | 6236.822 |  | 1.21544184 | 0.898430777 |
| FasL | 2511.256 | 6072.714 | 1543.04 |  | 2.418197906 | 0.614449594 |
| HSP27 | 10771.12 | 11780.67 | 9662.651 |  | 1.093727486 | 0.897088789 |
| HSP60 | 7827.259 | 10940.17 | 7067.666 |  | 1.39770129 | 0.902955428 |
| HSP70 | 5923.908 | 10645.79 | 6624.068 |  | 1.797089016 | 1.118192247 |
| HTRA | 5130.092 | 11104.5 | 7008.473 |  | 2.164581064 | 1.366149574 |
| IGF-I | 2565.614 | 6512.648 | 1665.227 |  | 2.538436413 | 0.649055937 |
| IGF-II | 6887.333 | 9403.676 | 6520.723 |  | 1.365358115 | 0.946770397 |
| IGFBP-1 | 1424.979 | 2728.322 | 1145.611 |  | 1.914640146 | 0.803949392 |
| IGFBP-2 | 2965.23 | 4545.817 | 1481.614 |  | 1.53304027 | 0.499662421 |
| IGFBP-3 | 2636.218 | 5171.068 | 1717.993 |  | 1.961547945 | 0.651688517 |
| IGFBP-4 | 1159.592 | 2487.825 | 670.9786 |  | 2.145431324 | 0.578633347 |
| IGFBP-5 | 5217.354 | 7078.417 | 2953.615 |  | 1.356706292 | 0.566113589 |
| IGFBP-6 | 2716.655 | 6117.988 | 1737.834 |  | 2.252029794 | 0.639696244 |
| IGF-1sR | 2347.122 | 5322.888 | 1517.432 |  | 2.267836099 | 0.646507467 |
| Livin | 2536.089 | 5521.888 | 1730.145 |  | 2.177324219 | 0.682209891 |
| p21 | 10927.87 | 12174.43 | 10321.5 |  | 1.114072001 | 0.944511602 |
| p27 | 4526.759 | 9240.434 | 6282.05 |  | 2.041291352 | 1.38775888 |
| p53 | 5802.145 | 10478.94 | 3976.637 |  | 1.806045868 | 0.685373599 |
| SMAC | 9599.084 | 12045.6 | 9701.23 |  | 1.254869735 | 1.010641224 |
| Survivin | 4534.712 | 6968.176 | 2943.527 |  | 1.536630331 | 0.649110021 |
| s TNF-R1 | 2794.698 | 6843.75 | 2871.169 |  | 2.44883347 | 1.027362885 |
| s TNF-R2 | 1473.286 | 3696.224 | 1206.887 |  | 2.508829922 | 0.81918039 |
| TNF-α | 281.7454 | 911.5815 | 400.3595 |  | 3.235479621 | 1.420997468 |
| TNF-β | 1507.432 | 4530.67 | 1709.507 |  | 3.005555143 | 1.134052481 |
| TRAILR-1 | 2838.668 | 6196.305 | 2276.098 |  | 2.182821309 | 0.801819022 |
| TRAILR-2 | 3727.589 | 7765.887 | 2588.827 |  | 2.083353878 | 0.69450441 |
| TRAILR-3 | 3812.707 | 6913.755 | 2498.596 |  | 1.813345479 | 0.655333861 |
| TRAILR-4 | 2820.955 | 6358.302 | 1927.681 |  | 2.253953714 | 0.683343294 |
| XIAP | 3981.875 | 8177.259 | 4895.372 |  | 2.053620217 | 1.229413781 |

Protein levels more than 2-fold was considered as the cut-off values.
